# Multiclass Classification Based on Combined Motor Imageries

**DOI:** 10.3389/fnins.2020.559858

**Published:** 2020-11-19

**Authors:** Cecilia Lindig-León, Sébastien Rimbert, Laurent Bougrain

**Affiliations:** ^1^Université de Lorraine, CNRS, LORIA, Inria, Nancy, France; ^2^Faculty of Engineering, Computer Science and Psychology, Institute of Neural Information Processing, Ulm University, Ulm, Germany

**Keywords:** brain-computer interface (BCI), combined motor imageries, multilabel classification, common spatial pattern (CSP), electroencephalography (EEG)

## Abstract

Motor imagery (MI) allows the design of self-paced brain–computer interfaces (BCIs), which can potentially afford an intuitive and continuous interaction. However, the implementation of non-invasive MI-based BCIs with more than three commands is still a difficult task. First, the number of MIs for decoding different actions is limited by the constraint of maintaining an adequate spacing among the corresponding sources, since the electroencephalography (EEG) activity from near regions may add up. Second, EEG generates a rather noisy image of brain activity, which results in a poor classification performance. Here, we propose a solution to address the limitation of identifiable motor activities by using combined MIs (i.e., MIs involving 2 or more body parts at the same time). And we propose two new multilabel uses of the Common Spatial Pattern (CSP) algorithm to optimize the signal-to-noise ratio, namely MC2CMI and MC2SMI approaches. We recorded EEG signals from seven healthy subjects during an 8-class EEG experiment including the rest condition and all possible combinations using the left hand, right hand, and feet. The proposed multilabel approaches convert the original 8-class problem into a set of three binary problems to facilitate the use of the CSP algorithm. In the case of the MC2CMI method, each binary problem groups together in one class all the MIs engaging one of the three selected body parts, while the rest of MIs that do not engage the same body part are grouped together in the second class. In this way, for each binary problem, the CSP algorithm produces features to determine if the specific body part is engaged in the task or not. Finally, three sets of features are merged together to predict the user intention by applying an 8-class linear discriminant analysis. The MC2SMI method is quite similar, the only difference is that any of the combined MIs is considered during the training phase, which drastically accelerates the calibration time. For all subjects, both the MC2CMI and the MC2SMI approaches reached a higher accuracy than the classic pair-wise (PW) and one-vs.-all (OVA) methods. Our results show that, when brain activity is properly modulated, multilabel approaches represent a very interesting solution to increase the number of commands, and thus to provide a better interaction.

## 1. Introduction

Motor imagery (MI) is a mental process during which subjects imagine themselves performing a movement without executing it, specifically by activating the haptic sensations (i.e., tactile, proprioceptive, and kinesthetic) felt during a real movement (Jeannerod, 1995; Guillot et al., 2009). Considering that MI consists of evoking a motor action, such a mental process activates the primary motor cortex and the additional motor areas in the same way as a real movement (Hétu et al., 2013). This activity can be analyzed by using electroencephalography (EEG) recordings, where rhythmic macroscopic oscillations with spectral peaks over the post-central somatosensory cortex around 10 Hz and over the precentral motor cortex at 20 Hz are observed (Jasper, 1936; Hari and Salmelin, 1997). These oscillations produce specific patterns of event-related desynchronization (ERD; i.e., a reduction of the oscillatory activity with respect to a resting period) and event-related synchronization (ERS; i.e., an increase in the oscillatory activity) within the mu/alpha (7–13 Hz) and beta (15–35 Hz) bands over the region associated with the body part engaged in the task (Pfurtscheller and Aranibar, 1979; Pfurtscheller and Neuper, 2001). More precisely, before and during an MI, ERD patterns appear gradually in the mu/alpha and beta bands Pfurtscheller and Lopes da Silva (1999), whereas at the end of the MI, ERS patterns are typically observed in the beta band (Pfurtscheller, 2001), and occasionally, in the mu band (Lindig-Leon et al., 2015).

For discriminating MIs involving different body parts, there are two particular considerations: (i) the lateralization during the activation of the motor cortex and (ii) the focal ERD/surround ERS effect. The mentioned lateralization implies that MIs executed by one side of the body activate the opposite side of the motor cortex. Thus, an MI of the right hand induces ERD patterns in the left side of the sensorimotor cortex, while an MI of the left hand appears in the right side (Pfurtscheller and Neuper, 1997, 2001; Neuper and Pfurtscheller, 2001; Neuper et al., 2009). In this way, the recognition of an MI is based on the location over the motor cortex of the ERD patterns associated with the body part that is engaged in the task (Pfurtscheller, 2001; Blankertz et al., 2006; Blankertz et al., 2008b; Lotte et al., 2007; Müller-Putz et al., 2016). In addition, the focal ERD/surround ERS effect, which consists of the ERS patterns that are simultaneously found in the ipsilateral side of the motor cortex, also provide an insight into the body part that is engaged in the task (Suffczynski et al., 1999; Pfurtscheller, 2003; Jäncke et al., 2006). One hypothesis suggests that the focal ERD/surround ERS is a response to the selective attention given to a particular body part during a single MI (i.e., only one body part engaged in the motor task). For instance, an MI of the right hand elicits an ERD over the contralateral side (electrode C3), while inducing an ERS over the ipsilateral and central parts of the motor cortex (electrodes Cz and C4, which correspond to regions associated with the feet and left hand) (Pfurtscheller et al., 1993; Pfurtscheller, 2003). In the case of combined MIs (i.e., two or more body parts engaged at the same time), the focal ERD/surround ERS represents an interesting and still open question, considering that multiple body parts may be simultaneously engaged in the motor task and the associated ERD patterns might cancel out the ERS elicited by other sources.

Given that no stimulation is required to produce MIs, such a paradigm allows designing self-paced brain–computer interfaces (BCIs), which provides users with the freedom to send commands on demand (Mason et al., 2006). Consequently, and in contrast to other BCI paradigms that are restricted to a predefined time frame, MI-based BCIs can potentially afford an intuitive and continuous interaction (Wolpaw and Wolpaw, 2012). Therefore, MI represents an interesting solution to control neuroprostheses. However, considering the difficulty to afford multiple commands for EEG-based BCIs, a full interaction is still a challenge. Over the past decade, impressive improvements have been made for decoding complex motor activities from intracranial electrodes (Wodlinger et al., 2014; Yin et al., 2014; Tyson et al., 2015), with which it is possible to extract multiple mental states (i.e., control commands). Yet, despite the benefits of such a framework, complex EEG-based MIs have not been extensively studied and very little is known about their suitability for this purpose. In the present study, we investigate the use of combined MIs (Royer et al., 2013; Yi et al., 2013), which in contrast to the standard paradigm considerably increases the number of classes while using the same number of body parts (2^*n*^ compared to *n*, where *n* is the number of body parts, and when all possible combinations are considered). In general, the activity sources are chosen to cover a relatively large area over the sensorimotor cortex, while maintaining an adequate spacing among them to avoid mixing up specific activity. Consequently, given the distribution of sources along the sensorimotor cortex most of the MI-based BCIs are designed to identify ERD/ERS patterns generated by the left hand, right hand, and/or feet. Under a single label approach, using the three aforementioned activity sources allows designing a BCI with only three commands for interaction, which remains limited for designing efficient systems. On the contrary, with a multilabel approach we have designed a paradigm including the single and combined use of the left hand, right hand, and both feet together which, in addition to the rest condition, provide eight different classes (rest, left hand, feet, left hand and feet, right hand, both hands, right hand and feet, and both hands and feet).

In the present work, we propose a solution to address the limited number of identifiable activity sources, and we provide two new multilabel uses of the Common Spatial Pattern (CSP) algorithm. The CSP algorithm is very convenient, since it can be applied to any MI-based BCI while favoring high classification performances, it is also easy to implement and computationally efficient. However, given its formulation, CSP is constrained to binary problems. Consequently, the most common way to extend CSP to the multiclass case consists of solving a set of binary subproblems, either in a pair-wise (PW) or a one-vs.-all (OVA) approach. The main drawback to this solution is that the number of classifiers increases significantly with the number of classes, given that for a *k*-class problem the PW and OVA approaches require *k*(*k* − 1)/2, and *k* classifiers, respectively.

Here, we address the question of whether the EEG activity elicited during combined MIs can be analyzed independently over the sources related to each one of the body parts included in the paradigm to subsequently predict the class label from the combination of the extracted information. In this way, one can transform the original 8-class problem into three binary problems (i.e., one problem associated with each body part). In other words, one can convert a 2^*n*^-problem into *n* binary problems, where *n* is the number of body parts. Importantly, this transformation allows to apply the CSP algorithm to each one of the three binary problems and to obtain signals that are optimally discriminative with respect to variance. The obtained results show that this simplification is convenient, and they confirm that characterizing a multilabel task as the superposition of the involved sources represents a plausible model. In particular, for subjects that were able to modulate their brain activity very efficiently, we could verify neurophysiological plausibility. In such cases, a multilabel approach represents a very interesting solution to control more robust systems.

The main novelty in our study is the development of an 8-class multilabel paradigm, and its simplification based on the separation of sources. In the following, we describe the experimental paradigm of an 8-class multilabel paradigm combining right hand, left hand, and feet MIs. In section 3, we first present the theoretical framework for the feature extraction based on the CSP algorithm and we introduce the two new multilabel approaches. The first one of these methods, namely MC2CMI, generates three binary problems in which all MIs engaging one of the three selected body parts are grouped together in one class, and all MIs that do not engage the same body part are grouped together in the second class. In this way, for each binary problem the CSP algorithm produces features for determining if the given body part is engaged in the task or not. The second method, namely MC2SMI, operates in a very similar way, with the only difference that any of the combined MIs is considered during the training phase, which drastically accelerates the calibration time. In addition, we describe the classic multiclass methods named PW and OVA. In section 4, we show that both multilabel approaches outperform the classic solutions.

## 2. Materials

### 2.1. Participants

Seven right-handed healthy subjects (2 females, aged 31.8 ± 8.7 years) were recruited for this study. Subjects had no medical history that could have influenced the task (i.e., diabetes, antidepressant treatment, or neurological disorders). The experiment followed the statements of the WMA declaration of Helsinki on ethical principles for medical research involving human subjects World Medical Association (2002) and has been approved by the local ethical committee of Inria (COERLE, approval number: 2016-011/01) as it satisfied the ethical rules and principles of the institute.

### 2.2. Experimental Paradigm

**Figure 1:**
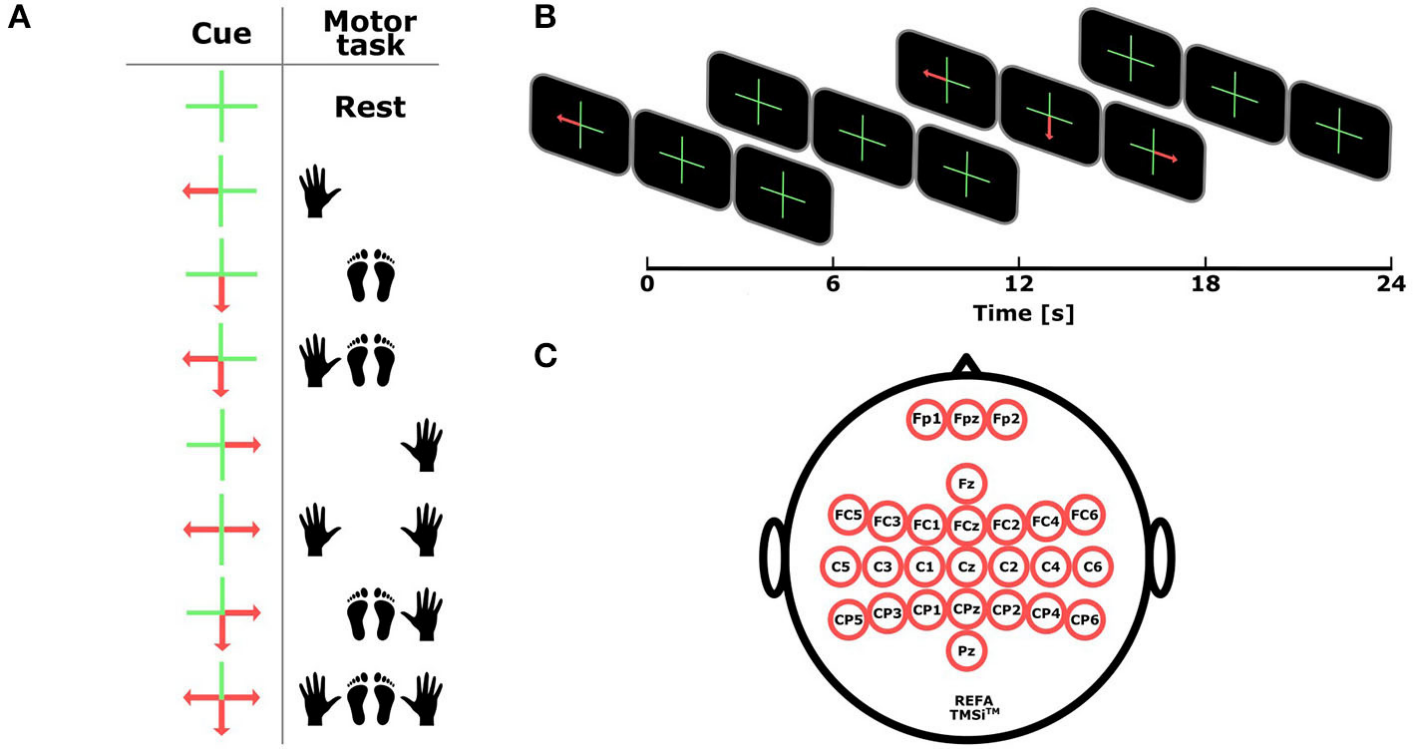
Experimental setup. **(A)** Task cue for each motor task. **(B)** Time scheme. Each trials lasts 12 s, the task cue is shown during the first 6 s, followed by a pause period of 6 s. **(C)** Distribution of the 26 considered electrodes mainly over the motor cortex.

Subjects were seated in a comfortable chair with the arms at their sides in front of a screen showing the task cue to be performed, which consisted of one of the eight mental states that it is possible to generate with all the combinations including the right hand, left hand, and both feet together, i.e., rest, left hand, feet, left hand and feet, right hand, both hands, right hand and feet, and both hands and feet (see Figure 1A). Subjects were instructed to imagine the opening/closing of their hands (with special attention over the thumbs due to the long distance between the feet and opposite thumb motor regions), and to imagine a fast up/down movement of their feet.

The whole session consisted of four runs, containing each one 10 trials per task for a total of 40 trials per class (320 trials considering the eight classes). For stimulus presentation, we used three panels that were simultaneously displayed on the screen, each of which was associated from left to right, to the left hand, feet, and right hand (see Figure 1B). Each trial was randomly presented and lasted for 12 s, starting at second “0” with a cross at the center of each panel and an overlaid arrow indicating for the next 6 s the motor task to be performed: an arrow pointing to the left side on the left panel for left hand, an arrow pointing down on the central panel for feet, an arrow pointing to the right side on the right panel for right hand, and the simultaneous presentation of these arrows for the corresponding combined MIs. The rest condition was indicated by the absence of arrows. After second 6, the task cue disappeared and the crosses were remaining for the next 6 s indicating the pause period before the next trial started.

### 2.3. EEG Recording

EEG signals were recorded at 256 Hz using a commercial amplifier Refa developed by TMS International™. Both the signal acquisition and the stimulation process were implemented on the OpenViBE platform^1^ (Renard et al., 2010). The EEG cap was fitted with 26 electrodes, namely, Fp1, Fpz, Fp2, Fz, FC5, FC3, FC1, FCz, FC2, FC4, FC6, C5, C3, C1, Cz, C2, C4, C6, CP5, CP3, CP1, CPz, CP2, CP4, CP6, and Pz, re-referenced with respect to the common average reference across all channels and located over the extended international 10–20 system positions to cover the primary sensorimotor cortex (see Figure 1C). Signals were band-pass filtered within the frequency range (8–30 Hz) using a fifth-order Butterworth filter.

## 3. Methods

In our experiment, we have focused on the activity generated by the left hand, right hand, and feet MIs. Thus, subjects activity is expected to be observed over three main regions. For the left hand, the corresponding source is located on the right hemisphere around electrode C4, whereas the right hand activates regions in the opposite side around electrode C3. In the case of the feet, both the left and right foot meet over central regions located around electrode Cz (see Figure 1). The following subsections present our framework for feature extraction based on the characterization of the brain activity over each one of these regions.

### 3.1. Feature Extraction

As a result of the volume conduction, EEG signals generate a rather noisy image of brain activity, which results in a poor classification performance that worsens as the number of classes increases. In consequence, spatial filters are particularly effective to recover the significant information that is dispersed over different channels, and thus to generate discriminative features. This kind of filters can be fixed beforehand considering the sensor geometry and neurophysiological insights (e.g., Laplacians, bipolar) (Wolpaw and McFarland, 2004), or they can be optimized by using subject-specific training data (Guger et al., 2000; Blankertz et al., 2007, 2008a). Such is the case of the CSP method, a very popular algorithm in BCI research (Koles et al., 1990; Blankertz et al., 2008b; Lotte, 2014).

#### 3.1.1. Spatial Filtering: CSP Algorithm

The CSP algorithm is one of the most popular and efficient approaches for analyzing oscillatory activity (Koles et al., 1990; Blankertz et al., 2008b). Basically, the CSP algorithm generates a series of spatial filters that decompose multidimensional data into a set of uncorrelated components. These filters aim at extracting elements that simultaneously maximize the variance of one class, while minimizing the variance of the other one (Ramoser et al., 2001). Since the variance of band-pass filtered signals corresponds to band-power, this approach produces band power features with values that are maximally different between classes. This way the CSP algorithm achieves an efficient discrimination of mental states that are introduced by ERD/ERS activity (Pfurtscheller and Lopes da Silva, 1999).

Let us consider the mean of the normalized covariance matrices ${\bar{\Sigma }}_{k}$ of the *N* successive training trials for each class *k* as:

(1) $$\begin{array}{l} {\bar{\Sigma }}_{k}=\frac{1}{N}\overset{N}{\underset{i=1}{\sum }}\frac{{\text{E}}_{k,i}{\text{E}}_{k,i}^{⊤}}{\text{trace}\left({\text{E}}_{k,i}{\text{E}}_{k,i}^{⊤}\right)}, \end{array}$$

where ${\text{E}}_{k,i}\in {\mathbb{R}}^{C\times T},k\in \left\{1,2\right\}$ denotes the *i*th EEG trial belonging to class *k* recorded over *C* channels and *T* samples, with ⊤ representing the transpose operator. The spatial filters **W** can be obtained by solving the generalized eigenvalue decomposition problem that simultaneously diagonalize the mean covariance matrices of both classes

(2) $$\begin{array}{l} {\bar{\Sigma }}_{1}\text{W}=\Lambda {\bar{\Sigma }}_{2}\text{W}, \end{array}$$

where Λ represents the diagonal matrix of eigenvalues for Σ_1_. The spatial filtered signal *Y*_*k,i*_ can be obtained from the EEG trials *E*_*k,i*_ as:

(3) $$\begin{array}{l} {\text{Y}}_{k,i}={\text{W}}^{⊤}{\text{E}}_{k,i}. \end{array}$$

There are as many CSP filters as channels in the EEG signal, and each one of them is represented by a column vector of **W**. These filters are paired and not all of them are relevant for discrimination. Thus, after sorting all λ values, only the first *m* and the last *m* columns of **W** are selected. In the present work, for all methods and subjects *m* = 3 pairs of filters were considered (Blankertz et al., 2008b).

#### 3.1.2. Features

The selected feature vectors **v**_*i*_ generate the spatial filters coefficient matrix $\tilde{\text{W}}$, from which the *m* pairs of CSP features of the *i*th trial for the band-pass filtered EEG measurements can be computed as

(4) $$\begin{array}{l} {\text{v}}_{i}=\text{log}\left(\frac{\text{diag}\left({\tilde{\text{W}}}^{⊤}{\text{E}}_{i}{\text{E}}_{i}^{⊤}\tilde{\text{W}}\right)}{\text{trace}\left({\tilde{\text{W}}}^{⊤}{\text{E}}_{i}{\text{E}}_{i}^{⊤}\tilde{\text{W}}\right)}\right). \end{array}$$

### 3.2. Multilabel Approaches

#### 3.2.1. MC2CMI

**Figure 2:**
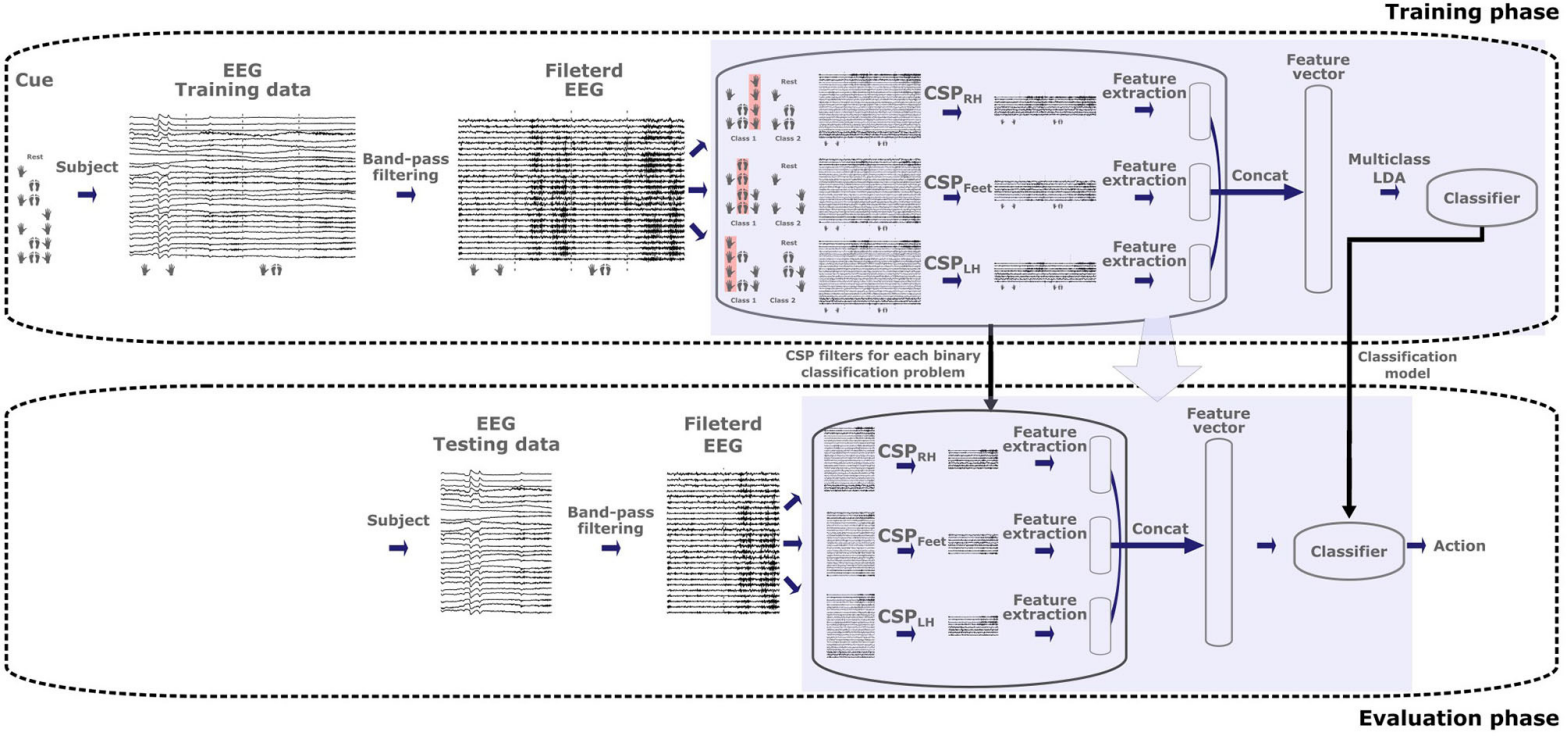
Architecture of the MC2CMI algorithm for the training and evaluation phases. The band-pass filtered EEG trials within the training data set are used to generate the three sets of Common Spatial Pattern (CSP) filters and the linear discriminant analysis (LDA) model of the MC2CMI method, both of which are subsequently applied to the testing data set.

The *8-class classifier trained on multilabel CSP features obtained from combined MIs* (MC2CMI) method, as illustrated in Figure 2, simplifies the original 8-class problem by transforming it into a set of 3 binary problems, each one concerning one of the activity sources associated with the body parts included in the paradigm (i.e., left hand, right hand, and feet), to determine whether they are engaged in an MI or not. During the training, the band-pass filtered EEG trials of the training data set are separated by grouping together all MIs including one specific body part in one class (hereinafter referred to as class 1), and all MIs that do not include it in the second class (class 2). Thus, the 2 classes are arranged for each binary problem as follows:

(*i*) Left hand:
  – Class 1: left hand, left hand and feet, both hands, and both hands and feet;
  – Class 2: rest, feet, right hand, right hand, and feet.
(*ii*) Feet:
  – Class 1: feet, left hand and feet, right hand and feet, and both hands and feet;
  – Class 2: rest, left hand, right hand, both hands.
(*iii*) Right hand:
  – Class 1: right hand, both hands, right hand and feet, and both hands and feet;
  – Class 2: rest, left hand, feet, left hand, and feet.

In this way, the CSP method can be applied directly to each one of the binary problems (see section 3.1.1). As mentioned, only three pairs of CSP filters are considered, and thus each binary problem generates features within a 6-dimensional space. All these features are subsequently concatenated together to generate the final feature vectors defined in an 18-dimensional space, where an 8-class linear discriminant analysis (LDA) model is trained over the eight classes. Finally, during the validation phase, the band-pass filtered EEG trials of the testing data set are mapped into the classification space in order to predict the corresponding class labels.

#### 3.2.2. MC2SMI

**Figure 3:**
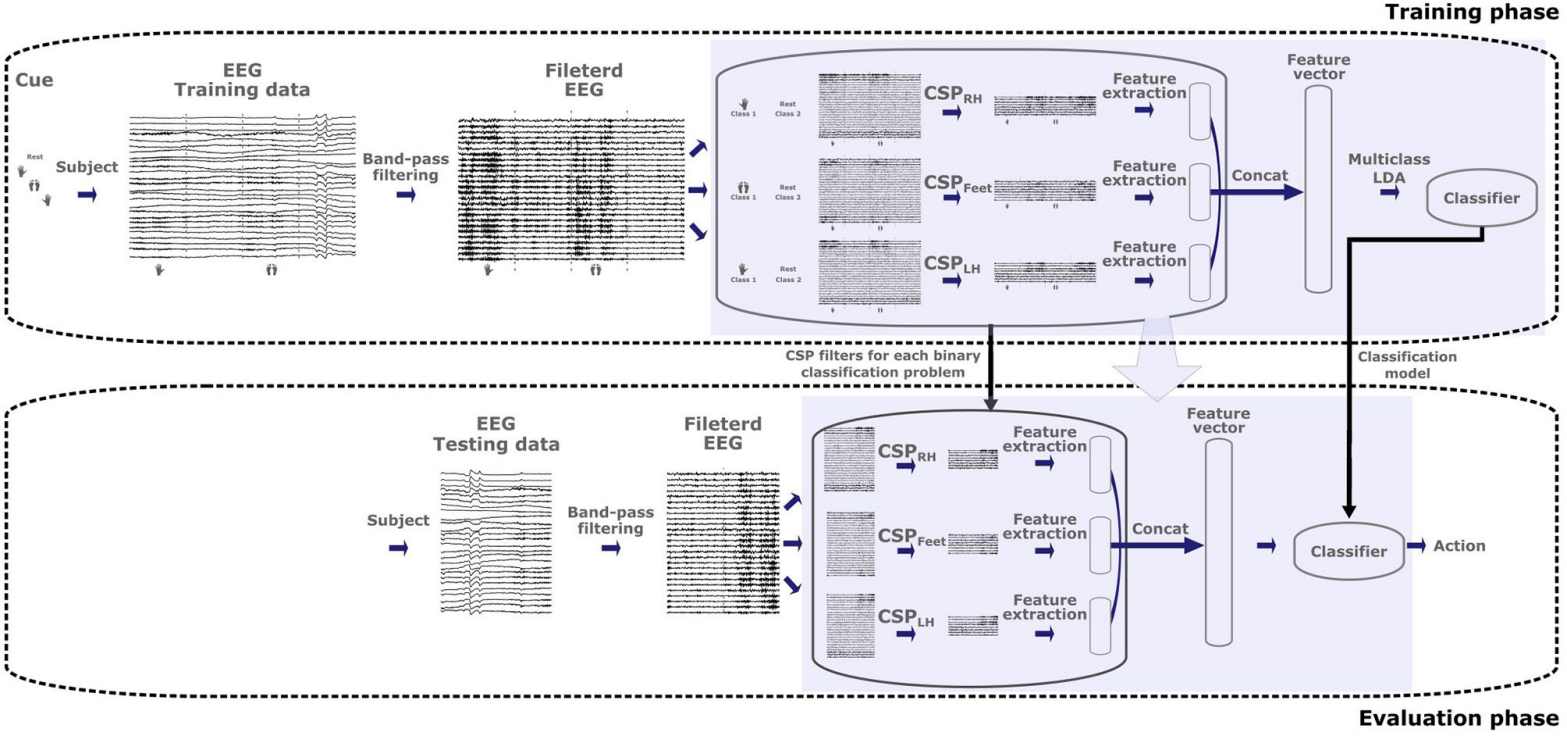
Architecture of the MC2SMI algorithm for the training and evaluation phases. The band-pass filtered EEG trials within the training data set are used to generate the three sets of Common Spatial Pattern (CSP) filters and the linear discriminant analysis (LDA) model of the MC2SMI method, both of which are subsequently applied to the testing data set.

One simple question that we address is whether it is possible to train the classification model by using training data only from single MIs, which would considerably reduce the calibration time of the system. Contrarily to the classic multiclass extensions, the multilabels approaches allow to infer the features for combined MIs from the superposition of features extracted independently over each source during single MIs. In the case of combined MIs, the feature vectors can be generated by adding the features obtained from the rest condition over sources that are not engaged in the motor task, and by adding the features obtained from single MIs of the body part(s) that is/are engaged in the task. In this way, we have evaluated a second version in which only single MIs are considered during the training phase, namely *8-class classifier trained on multilabel CSP features obtained from single MIs* (MC2SI) approach (see Figure 3). To this end, we make the assumption that combined MIs can be modeled as the superposition of the activity generated by each one of the involved body parts. Thus, during the training phase, the two classes in each one of the three binary problems are rearranged as follows:

(*i*) Left hand:
  – Class 1: left hand;
  – Class 2: rest.
(*ii*) Feet:
  – Class 1: feet;
  – Class 2: rest.
(*iii*) Right hand:
  – Class 1: right hand;
  – Class 2: rest.

As before, we consider the three most discriminant pairs of CSP filters by applying Equation (4), which produces features over six dimensions for each one of the three binary problems. In the same way, these features are subsequently concatenated into vectors over 18 dimensions to train an 8-class LDA model. Finally, the band-pass filtered EEG trials of the testing data set are mapped into the classification space in order to predict the corresponding class labels.

### 3.3. Classical Multiclass Methods

In order to compare the performance of the MC2CMI method to classic solutions, we also include the results obtained by the PW and OVA approaches.

#### 3.3.1. PW Approach

This approach consists of training *K*(*K* − 1)/2 binary classifiers for a K-multiclass problem. Each one of these binary classifiers is trained over the data points from a pair of classes in the original training set, and must learn to separate the two classes. For label prediction, all the *K*(*K* − 1)/2 classifiers are applied to the unknown data point, and the label is assigned by following a voting scheme where the class that got the highest number of predictions is selected (Bishop, 2006).

Considering the eight different MIs included in the paradigm, the PW approach requires 8*7/2 = 28 binary classifiers, each of which is trained over features defined in a 6-dimensional space corresponding to the projection of the first three pairs of CSP filters obtained by the discrimination of two different MIs.

#### 3.3.2. OVA Approach

This strategy involves training *K* binary classifiers for a K-multiclass problem. Each binary classifier is trained over all data points in the original training set, with the samples of the *K*_*i*_ class as positive samples and all other samples as negatives. This approach requires all *K* binary classifiers to generate a real-valued confidence score to make a decision, rather than just a class label, considering that class labels alone can lead to ambiguities, where multiple classes are predicted for a single data point. Even though this strategy is popular, it suffers from several problems. First, the scale of the confidence values may differ between the binary classifiers. Second, even if the class distribution is balanced in the training set, the binary classification learners see unbalanced distributions because typically the set of negatives they see is much larger than the set of positives (Bishop, 2006).

Considering the eight different MIs included in the paradigm, the OVA approach requires eight binary classifiers, each of which is trained over features defined in a 6-dimensional space corresponding to the projection of the first three pairs of CSP filters obtained by the discrimination of 1 MI against all the remaining ones.

### 3.4. Classification

After feature extraction, we have applied for all methods an LDA model fitted on the feature vectors and the corresponding training labels. The model assumes that the feature vectors present a Gaussian mixture distribution and that all classes have the same covariance matrix. The predicted label is then assigned according to the class that generates the minimum expected classification cost. We have applied a Box's M test to verify for equality of the covariance matrices, and even though it failed in some cases, we obtained better results than when using quadratic discriminant analysis (QDA), which allows the variation of the class covariance matrices. In this regard, QDA requires more parameters than LDA, i.e., the covariance matrices of all classes, which considerably increases the method variance. On the other hand, the assumption that all classes in our problem share a common covariance matrix does not cause an important bias.

The MC2CMI and MC2SMI methods concatenate the features that are generated by each one of the CSP modules, which allows using a single multiclass classification model. In contrast, the PW and OVA approaches use an LDA model for each one of the binary problems that are generated to solve the 8-class problem, i.e., 28 in the case of the PW method, and 8 for the OVA approach. In both cases, after evaluating all the binary classifications, the predicted label is assigned according to a vote scheme where the class summing the highest score is selected.

There are many classification techniques that can be applied in combination with the proposed methods. However, considering that both the MC2CMI and MC2SMI approaches consist of feature extraction methods for EEG combined MIs, we selected a simple classification model to evaluate the discriminative power of the generated features. In this way, the overall performance does not rely on the selection of multiple parameters when using more sophisticated classification techniques, which is out of the scope of this work.

## 4. Results

In our study, we investigated the possibility of decoding EEG signals recorded during motor tasks combining three body parts, i.e., left hand, right hand, and feet. We recorded seven subjects in a series of trials during which they had to generate the eight possible different mental states considering the imagined movement of these three body parts, i.e., rest, left hand, feet, left hand and feet, right hand, both hands, right hand and feet, and both hands and feet (see section 2.2). The research question in our study is to determine whether the ERD patterns in the EEG signals associated with one particular region remain consistent regardless the activation of other sources. To this end, we generated three binary problems in which the EEG signals were grouped into two classes: (i) all MIs including 1 of the 3 body parts in one class, and all the MIs that do not include it in the other class, for the CM2CMI method, and (ii) a single MI in one class, and the resting state in the other class, for the CM2SMI method.

### 4.1. ERD/ERS Modulations

**Figure 4:**
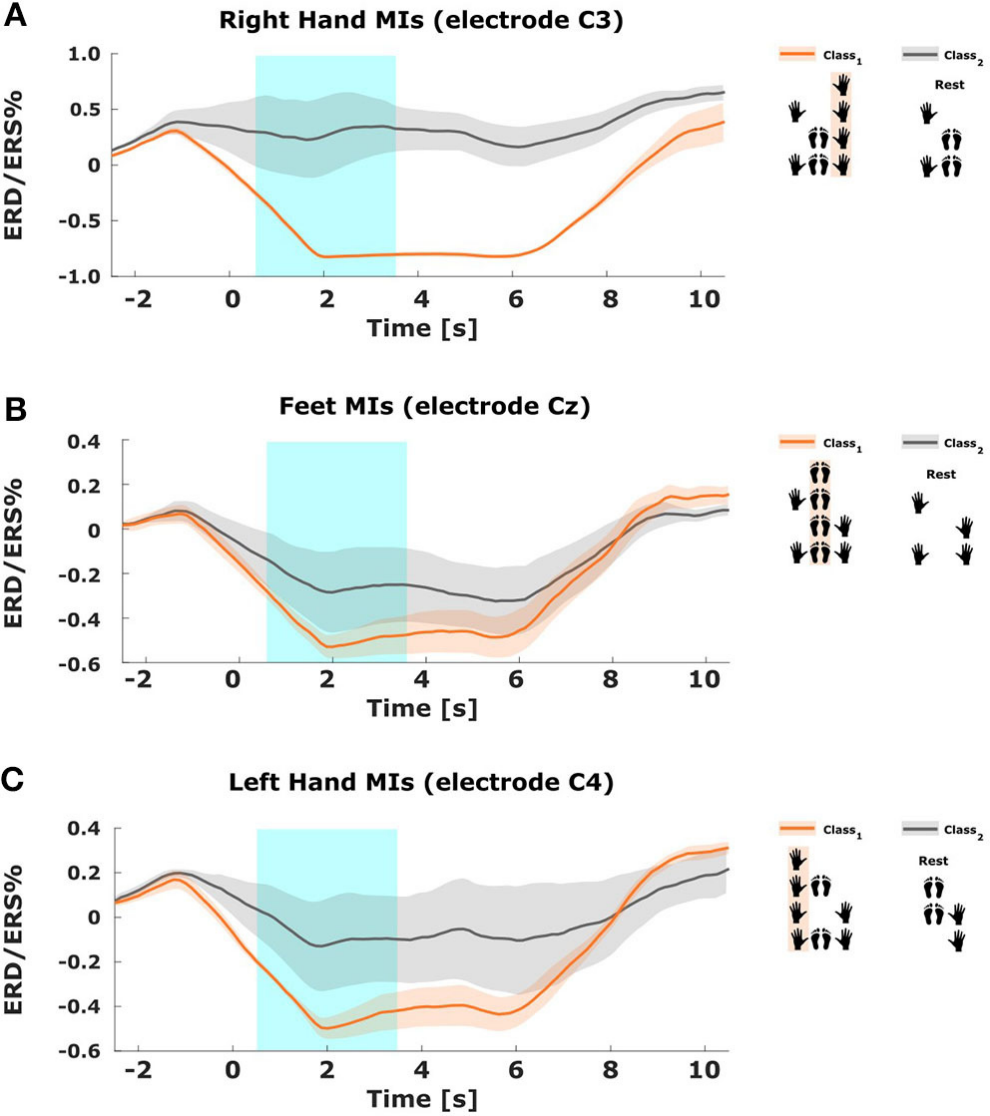
Modulations over the three main sources for subject 2. Each plot presents the mean ERS/ERD% patterns of the band-pass filtered EEG trials grouped within classes 1 and 2 for each one of the three modules in the CM2CMI method (solid lines). The shaded regions represent the standard errors of the mean, and the blue box within (0.5–3.5 s) indicates the time window that was considered for classification. **(A)** ERS/ERD% patterns in electrode C4 for class 1, i.e., motor imageries (MIs) including the left hand (orange line), and for class 2, i.e., MIs that do not include the left hand (gray line). **(B)** ERS/ERD% patterns in electrode Cz for class 1, i.e., MIs including the feet (orange line), and for class 2, i.e., MIs that do not include the feet (gray line). **(C)** ERS/ERD% patterns in electrode C3 for class 1, i.e., MIs including the right hand (orange line), and for class 2, i.e., MIs that do not include the right hand (gray line).

Figure 4 shows a schematic view of these arrangements (on the right side) and the resulting ERD/ERS% fluctuations over the main sources associated with each body part (on the left side) (see Supplementary Figure 1 for a complete topographic view across all subjects). In all cases, we observe the well-known ERD pattern over controlateral sources during an MI of the associated body part (orange lines). Furthermore, the variation caused by the activation of other sources during combined MIs remains low. In contrast, the modulations associated with MIs excluding the same body part (gray lines) present much higher values (ERS modulation). And as expected, considering that the elements within these groups are different combinations of MIs without a consistent pattern, the variations among the mean values are also stronger (see Supplementary Figures 2, 7 for other subjects).

**Figure 5:**
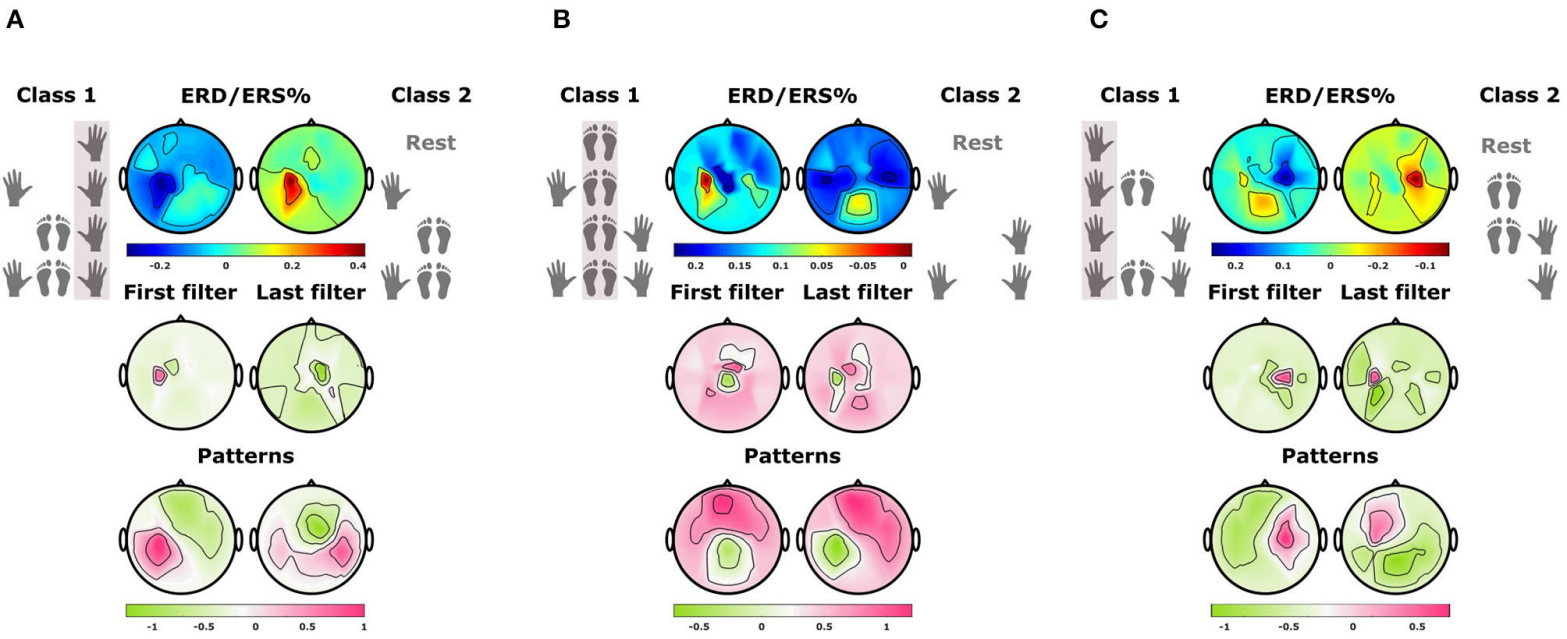
Common Spatial Pattern (CSP) modules in the MC2CMI method for subject 1. **(A)** The top topographies show the ERD/ERS% mean values over the selected time window of 0.5–3.5 s for classes 1 (including the right hand) and 2 (not including the right hand). The topographies in the bottom show the first and last filters of the CSP matrix trained over both classes, and the corresponding patterns. The same illustration is presented **(B)** for the MIs excluding/including the feet, and **(C)** for the MIs excluding/including the left hand.

From analyzing Figure 4, we found that a 3-s window starting 0.5 s after the cue was a convenient choice to find accentuated ERD patterns for all subjects. Therefore, we selected this period to train the CSP modules applied by the MC2CMI and the MC2SMI methods. Considering that the CSP filters generate subject-specific patterns, each method was trained independently for each subject. The mean covariance matrices ${\bar{\Sigma }}_{1}$ and ${\bar{\Sigma }}_{2}$ in Equation (2) are computed, respectively, by averaging the covariance matrices of classes 1 and 2 for each one of the three CSP modules, i.e., RH (right hand), FEET (feet), and LH (left hand). Figure 5 presents an example of the CSP analysis for each one of the three CSP modules of the MC2CMI method applied on data from subject 1. The topographic maps on top show the interpolation across all electrodes of the ERD/ERS% mean values for both classes. The smallest values shown in blue emerge again over the expected regions. Accordingly, we find correspondence of such neurophysiological insights when visualizing the CSP filter coefficients and their associated patterns (topographies below). In each case, we present two pairs of vectors (*w*_*j*_, *a*_*j*_) corresponding to the largest and the smallest eigenvalues, where *w*_*j*_ and *a*_*j*_ represent the *j*th columns of *W* and *A* = *W*^−1^, respectively (see section 3.1.1). On the other hand, the corresponding patterns show how the activity from different sources is projected onto the scalp, which can be used to verify the neurophysiological plausibility when finding strong weights over the corresponding motor cortex areas, as stated in the literature (Pfurtscheller and Neuper, 2001).

**Figure 6:**
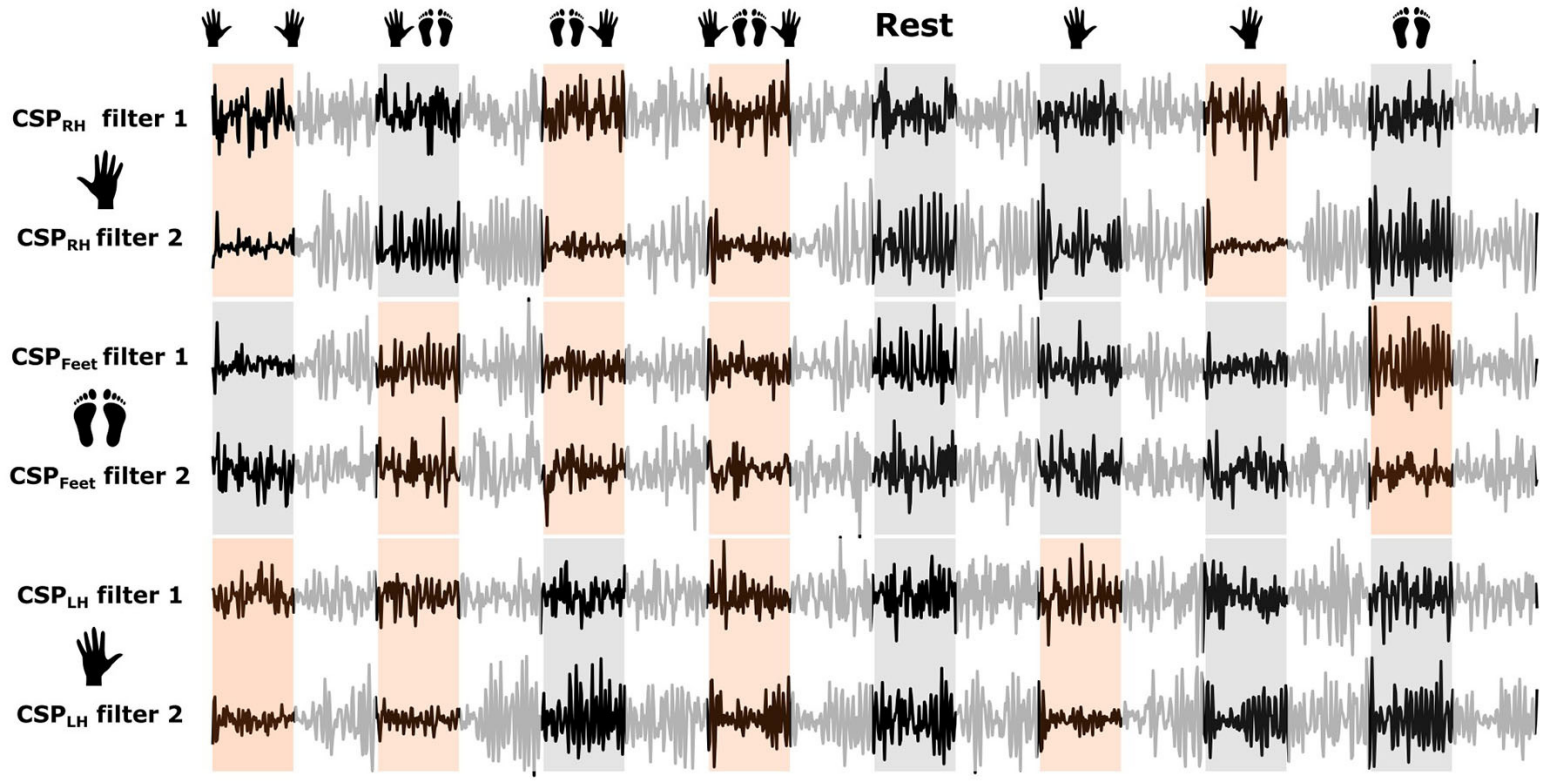
Effect of spatial Common Spatial Pattern (CSP) filtering. The graph shows continuous band-pass filtered EEG after applying the CSP filters. Those intervals during which a specific body part is engaged in the task are shaded orange and present smaller variance for the last CSP filter, whereas when using the same filter in segments where the same body part is not active (shaded gray), the variance is larger. We observe the opposite behavior for the first CSP filter. The regions shaded white represent the inactive periods during pauses.

**Figure 7:**
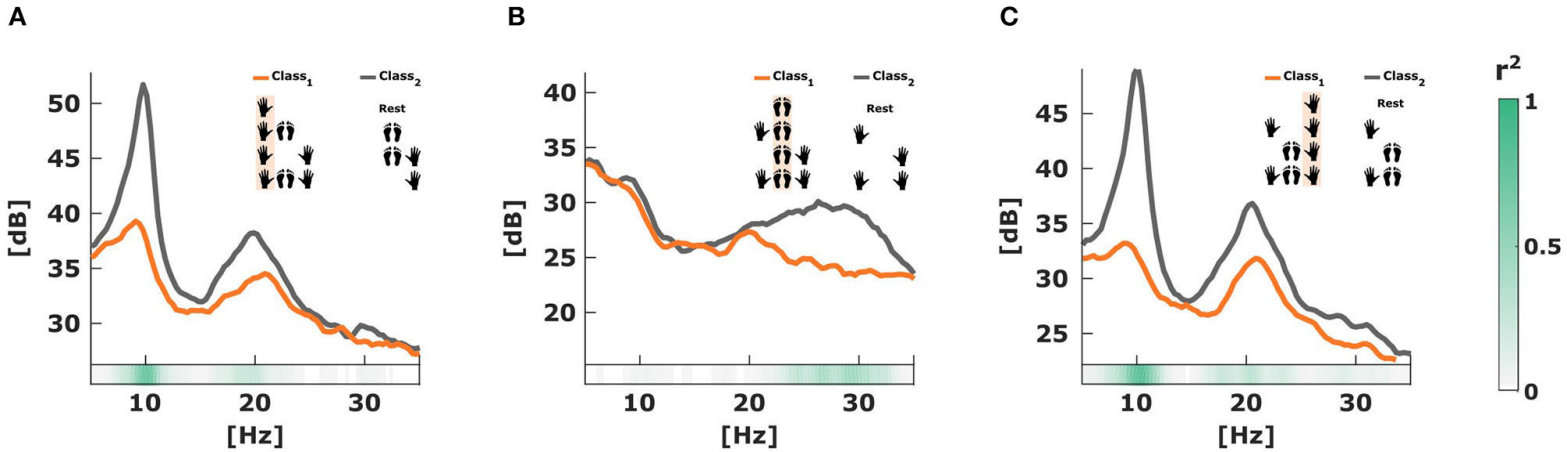
Spectra over the first Common Spatial Pattern (CSP) filter projection. **(A)** Problem associated with the left hand. **(B)** Problem associated with the feet. **(C)** Problem associated with the right hand. All plots are generated from the same dataset over the selected time window of 0.5–3.5 s but using different spatial filters. The discrimination between the two conditions is quantified by the *r*^2^-value.

The CSP filters project the band-passed filtered EEG data in order to generate signals that are optimally discriminative with respect to their variance. The effect of the CSP filters over the band-passed filtered EEG data of subject 2 when applying the MC2CMI method is shown in Figure 6. Here, we can observe the CSP projections using the largest and the smallest eigenvalues generated by each one of the three CSP modules of the MC2CMI method over a segment during which the subject performed each one of the eight MIs consecutively. In each module, there is a strong contrast in the variance among segments during which the corresponding body part is engaged in the motor task, and segments during which it is not engaged. Those intervals during which the specific body part is engaged in the task are shaded orange and present smaller variance along the last filter, whereas when using the same filter in segments where the same body part is not active (shaded gray), the variance is larger. Furthermore, in projections using the first filter we observe the opposite behavior, i.e., the variance is smaller along segments during which the specific body part is not used, and larger when the same body part is engaged. These changes in variance are optimal for discriminating mental states that are introduced by ERD/ERS activity. To verify the discriminative power of these projections, we have analyzed the power spectra of both classes in the frequency domain (see Figure 7), where we found spectral peaks around 12 Hz revealing a strong discriminative power.

### 4.2. Spectral Analysis

The changes in variance among the two classes characterize the ERD/ERS modulation observed during motor tasks, which can be used to generate effective features for discrimination. To quantify the discriminative power of the projected signals, we can analyze the two classes in the frequency domain. In Figure 7, we present a comparison between the spectra of the first CSP filter projections of both classes for each one of the three CSP modules in the MC2CMI method for subject 2. The difference in amplitude showing much lower values over those signals including a specific body part (orange lines), from those that do not include it (gray lines), demonstrates the discriminative power of the CSP filtering effect, which can be measured in terms of the *r*^2^ value (green color bars shown below).

**Figure 8:**
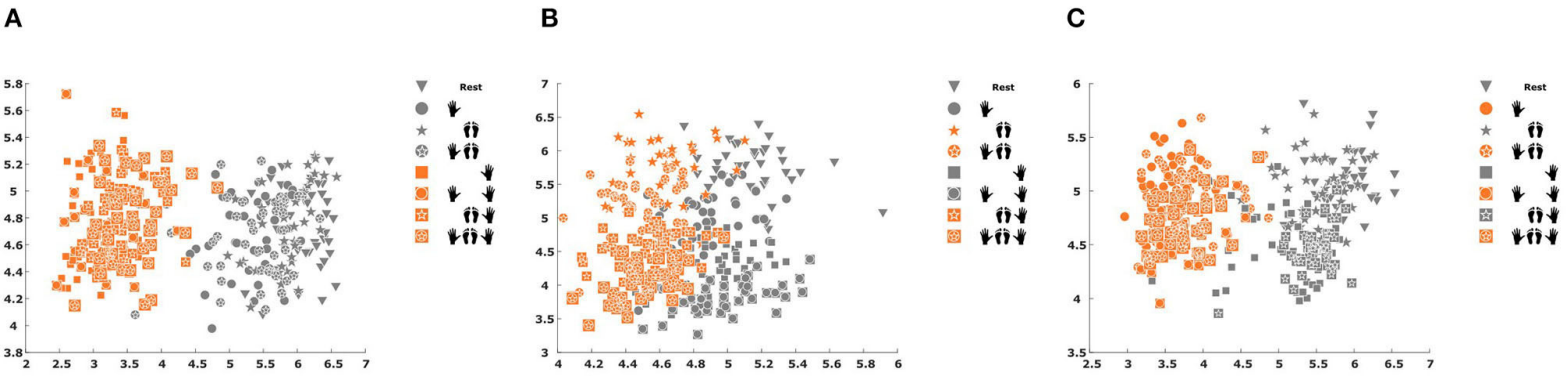
Features generated by each one of the three Common Spatial Pattern (CSP) modules in the MC2CMI method for subject 2. **(A)** Features from the RH module. **(B)** Features from the FEET module. **(C)** Features from the LH module. All plots are generated from the same dataset over the selected time window of 0.5–3.5 s but using different spatial filters.

Figure 8 shows an example of the features generated by each one of the three CSP modules in the MC2CMI method after applying Equation (4) using the first pair of CSP filters over data from subject 2. For visualization purposes, we show the results of using only the first pair of CSP filters, considering that, as mentioned in section 3.2.1, we used three pairs of filters to extract the CSP features in each module, so that each feature vector was defined within an 18-dimensional space after concatenating all components from all three binary problems. Note that the separability between both classes in the classification space is significant, specially for the problems associated with the left hand (Figure 8A) and the right hand (Figure 8C).

We used a 10-fold cross-validation scheme to evaluate the performance of each method. During each one of the 10 evaluations, four trials per class were randomly selected (without replacement) to build the testing set (32 trials in total), whereas the 36 remaining trials were used to generate the training set (288 trials in total). We used the same data partition to evaluate all methods to provide a fair comparison. The reached mean accuracy across subjects together with the standard error of the mean is presented in Table 1. In order to compare the performance of the MC2CMI and MC2SMI methods to the classic solutions, we also include the results obtained by the PW and OVA approaches. All methods were applied over the same band-pass filtered EEG data to provide a fair comparison. In addition to LDA, we have also applied support vector machines (SVMs) and decision trees for classification. Results using the MC2CMI method are superior than when using the classic approaches, however they do not outperform the accuracy reached by LDA classification, and in both cases further parameter optimization must be investigated. The results of using SVMs with linear kernel, and the results of using decision trees to evaluate all methods are shown in Supplementary Tables 1, 2, respectively.

**Table 1:** Classification accuracies.

**Subject**	**MC2CMI**	**MC2SMI**	**PW**	**OVA**
1	**51.25**	45.83	41.67	33.75
2	**82.81**	75.31	78.75	67.5
3	47.81	**51.88**	45.94	46.56
4	34.06	**39.38**	30.94	31.88
5	**47.81**	43.44	37.81	35.94
6	**55.63**	52.19	55.31	49.38
7	**65.63**	63.44	61.56	58.75
AVG	**55** ± **5.86%**	53.06 ± 4.72%	50.28 ± 6.14%	46.25 ± 5.09%

*Performance across all subjects after applying a 10-fold cross-validation procedure to assess the MC2CMI, together with the fast training version denoted as MC2SMI that was considered to optimize calibration times by training only over single MIs. We also present the results generated by the PW and the OVA approaches. All methods were applied over the same band-pass filtered EEG data to provide a fair comparison. The mean average across subjects is presented together with the standard error of the mean. For each subject, the best result is indicated by bold characters*.

**Figure 9:**
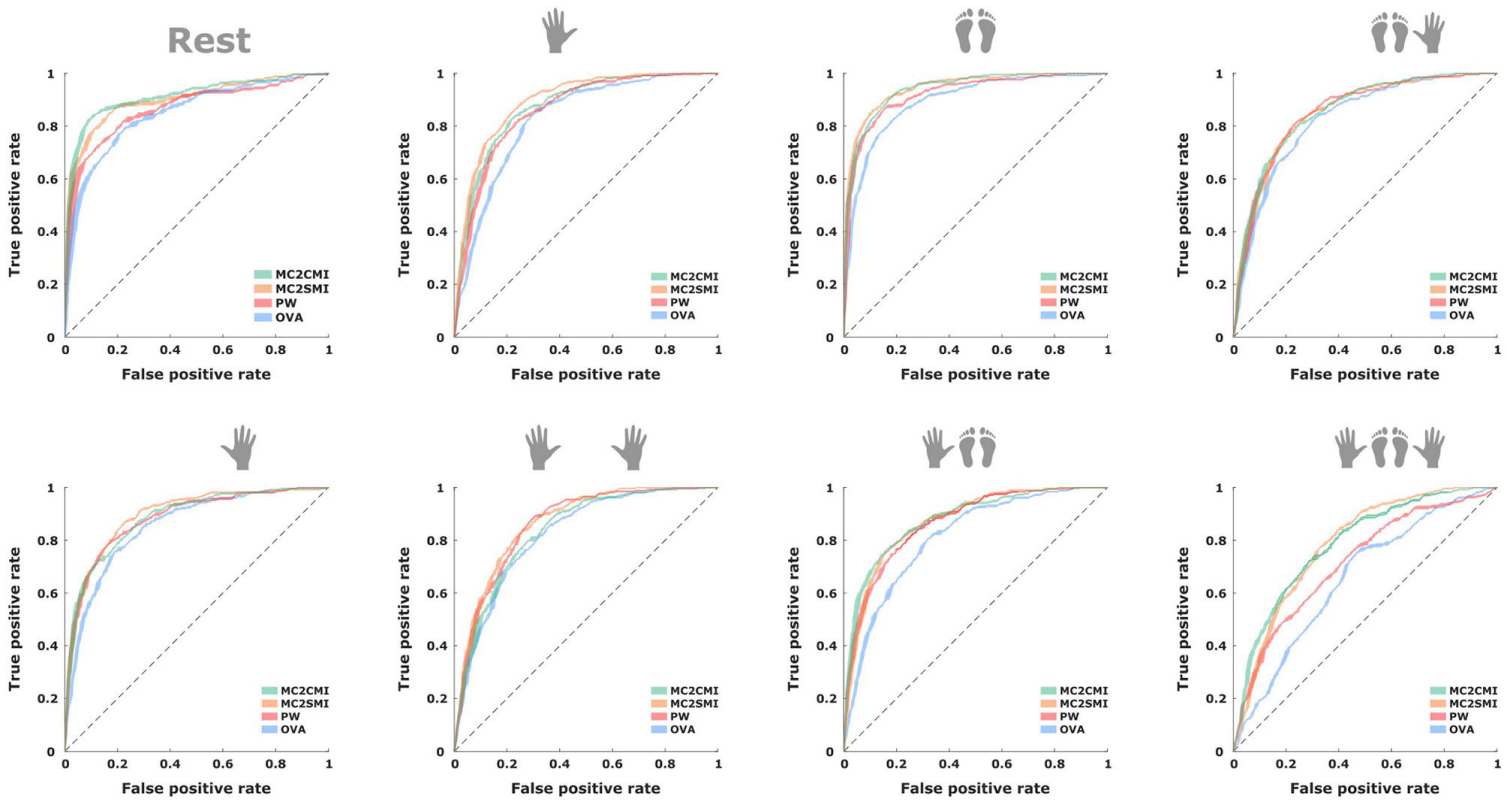
Mean across subjects of the receiver operating characteristic (ROC) curves for all classes and models. Each plot shows the ROC curves of one of the eight classes for the predictions generated by the MC2CMI, MC2SMI, PW, and OVA approaches.

Table 2 shows the *p*-values generated after applying a Wilcoxon rank sum test to verify that the results generated by each one of the presented methods are significantly different. As expected, in the case of the MC2CMI and MC2SMI approaches we do not find a significant difference, considering that the MC2SMI approach is only a simplification of the MC2CMI method. On the contrary, we found a strong evidence supporting that MC2CMI method is significantly different with respect to the PW and OVA approaches. In order to confirm that this difference represents an improvement on the classification task, we present the mean receiver operating characteristic (ROC) curves across subjects for each one of the eight classes generated after applying each method (see Figure 9). Note that, for multiclass problems, this analysis can be performed using a pairwise comparison, i.e., one class vs. all other classes (Hand and Till, 2001). Again for all classes, the MC2CMI and MC2SMI generate a larger area under the curve (AUC), which reveals a better performance in comparison to the classic approaches (see Table 3).

**Table 2:** *p*-Values after applying a Wilcoxon rank sum test to verify differences between independent groups.

	**MC2CMI**	**MC2SMI**	**PW**	**OVA**
MC2CMI		*p* = 0.23	*p* = 0.003	*p* = 3.05*e*^−8^
MC2SMI			*p* = 0.07	*p* = 1.35*e*^−5^
PW				*p* = 0.011

**Table 3:** Area under the curve (AUC).

	**MC2CMI**	**MC2SMI**	**PW**	**OVA**
AUC	87.12 ± 2.34	**87.67 ± 2.28**	85.37 ± 2.99	81.8 ± 3.4

*Average of the AUCs across the eight different classes and subjects with the corresponding error of the mean. The best result is indicated by bold characters*.

The MC2CMI method performed very efficiently and it reached an accuracy of 82.81% for subject 2. Furthermore, it outperforms both the PW and OVA methods by around 5 and 9%, respectively, achieving a higher accuracy for all subjects (see Table 1). In the same way, the CM2SMI method outperforms the PW and OVA methods for all subjects, which is of special interest considering that it does not require patterns of combined MIs to train the multiclass classifier.

These results are quite promising considering that most of the classifications scores are significant and higher than the chance level. According to Müller-Putz et al. (2008), Combrisson and Jerbi (2015), and Jeunet et al. (2018), and considering that we have eight different classes each comprising 40 trials, the chance level would be to 22.5%. Finally, it is worth mentioning that for our classification problem, the MC2CMI and MC2SMI approaches require only three feature extraction modules, in comparison to the 28 and eight modules required by the PW and OVA methods, respectively. In this regard, the proposed method not only outperforms the classic solutions, but also optimizes the classification process and reduces the calibration time.

## 5. Discussion

In this study, we have investigated the use of combined MIs to provide EEG-based BCI interaction with an extended number of commands. Indeed, despite the benefits of such a framework, combined MIs have not been extensively studied and very little is known about their suitability for this purpose. To address this gap, we have designed a paradigm including of the left hand, right hand, and feet, which together with the rest condition provide eight different mental states, i.e., rest, left hand, feet, left hand and feet, right hand, both hands, right hand and feet, and all MIs. We have argued that the EEG activity elicited during combined MIs can be analyzed independently over the sources related to each one of the body parts included in the paradigm to subsequently predict the class label from the combination of the extracted information. With this idea, we have contributed with two new feature extraction approaches, namely MC2CMI and MC2SMI methods, which to our best knowledge have not been considered before.

### 5.1. Neurophysiological Specificities of Combined Motor Imageries

Analyzing the EEG activity elicited by each one of the body parts separately presents a very important advantage, considering that this simplifies the problem and reduces it into a set of binary decisions, which allows to apply the CSP algorithm for feature extraction. We have demonstrated that this approach is plausible in a series of analyses in both the frequency and time domains. First, we have inspected the ERD/ERS modulations from the two classes considered by each one of the binary problems formulated by the MCM2CMI method (see Figure 4, and Supplementary Figures 2–7 for all subjects). In all cases, there is a strong contrast between both classes given that the class grouping all mental states using one of the body parts produces oscillations with an increased desynchronization over the associated activity source. Crucially, this behavior remains relatively consistent among all MIs within each class, regardless the activity generated by other active sources during combined MIs. The topographic maps showing ERD/ERS values in Figure 5 (top) (Supplementary Figures 8–13 for more subjects) provide a complete view across all electrodes. Here, we can see how the brain activity is distributed over the region associated with the body part considered by each one of the three modules. This region appears in blue colors indicating small values (ERD%) for the class grouping all MIs using one of the body parts, and it appears in red colors indicating high values (ERS%) for the other class grouping all MIs that do not include the same body part. Accordingly, the CSP patterns associated with the largest eigenvalue presented below activate the same region in the brain. This provides evidence to verify neurophysiological plausibility, and it confirms that grouping all mental states within these two classes represents an effective solution. Considering that the strongest discriminative components correspond with the common source among combined MIs for one class, and a combination of the other two sources for the second class, as shown by the patterns associated with the smallest eigenvalue.

Interestingly, it was possible to train the system only with data from single MIs without a significant loss of performance. This not only reduces the calibration time and subjects' fatigue, but it also provides evidence to support our multilabel model, where the activity generated by a combined MI corresponds to the superposition of the activity generated by each one of the involved sources.

### 5.2. Difficulties to Produce (Combined) MIs

The question of whether the use of combined motor imageries is a suitable solution for EEG-based BCIs does not have a categorical answer, and this is because it totally depends on the subjects and their ability to modulate their brain waves during the different mental states. First of all, we have to consider that this task might be too complex to perform. In fact, there is evidence showing that even a single MI is generally difficult to achieve (Guillot et al., 2009; McAvinue and Robertson, 2009). Such complexity leads to highly variable MI-based BCI performances (Dickhaus et al., 2009; Vidaurre and Blankertz, 2010; Ahn et al., 2018; Thompson, 2019), and in some cases the control of this kind of systems is completely ineffective. In particular, the combination of MIs considerably increases the difficulty of the task, since it requires higher coordination and concentration (Jeunet et al., 2016). The results presented in Table 1 show that some subjects (i.e., subject 4) had difficulties in producing suitable patterns for the different MIs. Conversely, when subjects manage to effectively modulate their brain activity (i.e., subject 4), complex solutions are highly recommended to gain control over multiple commands. In any case, multiclass paradigm represents a challenge that becomes more difficult as the number of classes increases. In this sense, it is important to design intuitive systems where the link between the mental commands and their associated label is not difficult to establish.

Interaction conditions, such as the usability of the BCI, feedback, and so on influence the performance of users, in particular by reducing their mental load (Grangeon et al., 2011; Di Rienzo et al., 2012; Talukdar et al., 2019). Subjects training becomes essential to improve the execution of combined MIs, and thus to achieve a good performance (Jeunet et al., 2016). An appropriate long-term training with efficient instruction and gradual difficulty (Lotte, 2012) is a promising way to improve multiclass BCI control. In addition, the emotional state can have a strong influence on EEG patterns. For instance, during the recording period subject 2 used to practice yoga and relaxation regularly. These activities have shown to improve BCI control (Cassady et al., 2014; Rimbert et al., 2019), which could have a greater impact on the classification performance than any other processing technique. Thus, if subjects achieve to modulate their brain oscillations and generate suitable patterns for classification, the multilabel approach represents a very appropriate solution to gain control over multiple commands.

### 5.3. Limitations and Future Work

In this study, we have validated all approaches using a database of seven healthy subjects, which represents a small sample size for rigorously evaluating the robustness of the presented methods. Moreover, even though two subjects reached an accuracy above 65%, the mean accuracy was rather poor, so it is still an open question whether this paradigm represents an effective solution to provide online control for a significant population. Therefore, a vast database of healthy subjects including a significant number of individuals that practice yoga and/or relaxation regularly must be investigated in future works.

## 6. Conclusion

This study contributes to enriching the limited knowledge of combined MIs to provide users with multiple commands for BCI interaction (Devlaminck et al., 2010; Meng et al., 2016). This approach has the advantage of considerably increasing the number of different brain states while using the same number of body parts (in order of 2^*k*^ compared to *k*, where *k* is the number of body parts and when all possible combinations are considered). The most common approaches have focused on the left hand, right hand, and both hands (LaFleur et al., 2013; Lindig-León and Bougrain, 2015a,b). Here, we include a third source (i.e., feet), with which it is possible to obtain eight different mental states (i.e., rest, left hand, feet, left hand and feet, right hand, both hands, right hand and feet, and both hands and feet). In a similar study (Yi et al., 2013), the paradigm included the same three body parts. However, each foot was used separately together with the opposite hand during combined MIs, and the class involving all MIs (i.e., left hand, right hand, and feet) was not included.

The novelty in our study is also the analysis for feature extraction, which is carried out separately over each activity source related to the three body parts included in the paradigm. With this simplification, we have contributed with two new methods, namely MC2CMI and MC2SMI. Both approaches are multiclass uses of the CSP algorithm for multilabel problems. Both methods outperform the classic PW and OVA approaches. Moreover, in comparison to the 28 [i.e., 2^*n*^(2^*n*^ − 1)/2, where *n* is the number of body parts], and 8 (i.e., 2^*n*^) classifiers required by the PW and OVA approaches, respectively, the multilabel methods require only 3 (i.e., *n*) feature extraction modules. Additionally, the MC2SMI is trained using data from only single MIs without a significant loss of performance, which considerably reduces the calibration time.

In general, subjects performance was low and only in a few cases results were satisfactory. In this regard, the inefficiency cannot be attributed to the feature extraction and/or classification methods. In fact, most of the subjects were not able to properly modulate their brain signals during the different motor tasks, so that features were not well-separated in the classification space. This problem requires special attention, considering that the plausibility of multilabel approaches might depend on the development of training strategies that are efficient in guiding subjects to generate suitable patterns for classification. If this modulation is appropriate and subjects generate discriminative signals, multilabel approaches represent a very interesting solution for designing systems with multiple commands to afford an intuitive and continuous interaction, such as for a full 3D control, which is of particular interest for the implementation of prosthetic devices.

## Data Availability Statement

The raw data supporting the conclusions of this article will be made available by the authors, without undue reservation.

## Ethics Statement

The studies involving human participants were reviewed and approved by Local ethical committee of INRIA (COERLE, approval number: 2016-011/01). The patients/participants provided their written informed consent to participate in this study.

## Author Contributions

CL-L and LB designed the experiment. CL-L and SR performed the experiments and analyzed the data. CL-L designed the feature extraction methods. LB supervised the project. CL-L, SR, and LB wrote the paper. All authors contributed to the article and approved the submitted version.

## Conflict of Interest

The authors declare that the research was conducted in the absence of any commercial or financial relationships that could be construed as a potential conflict of interest.

## Footnotes

^1^http://openvibe.inria.fr
