# Supplementary material for: Multiclass Classification Based on Combined Motor Imageries
Source: Front Neurosci. 2020 Nov 19;14:559858. doi: 10.3389/fnins.2020.559858 (PMC7710761; doi:10.3389/fnins.2020.559858)
Supplement: Supplementary file 1 [file Data_Sheet_1.pdf]

Supplementary Material:
Multiclass classification based on combined motor imageries

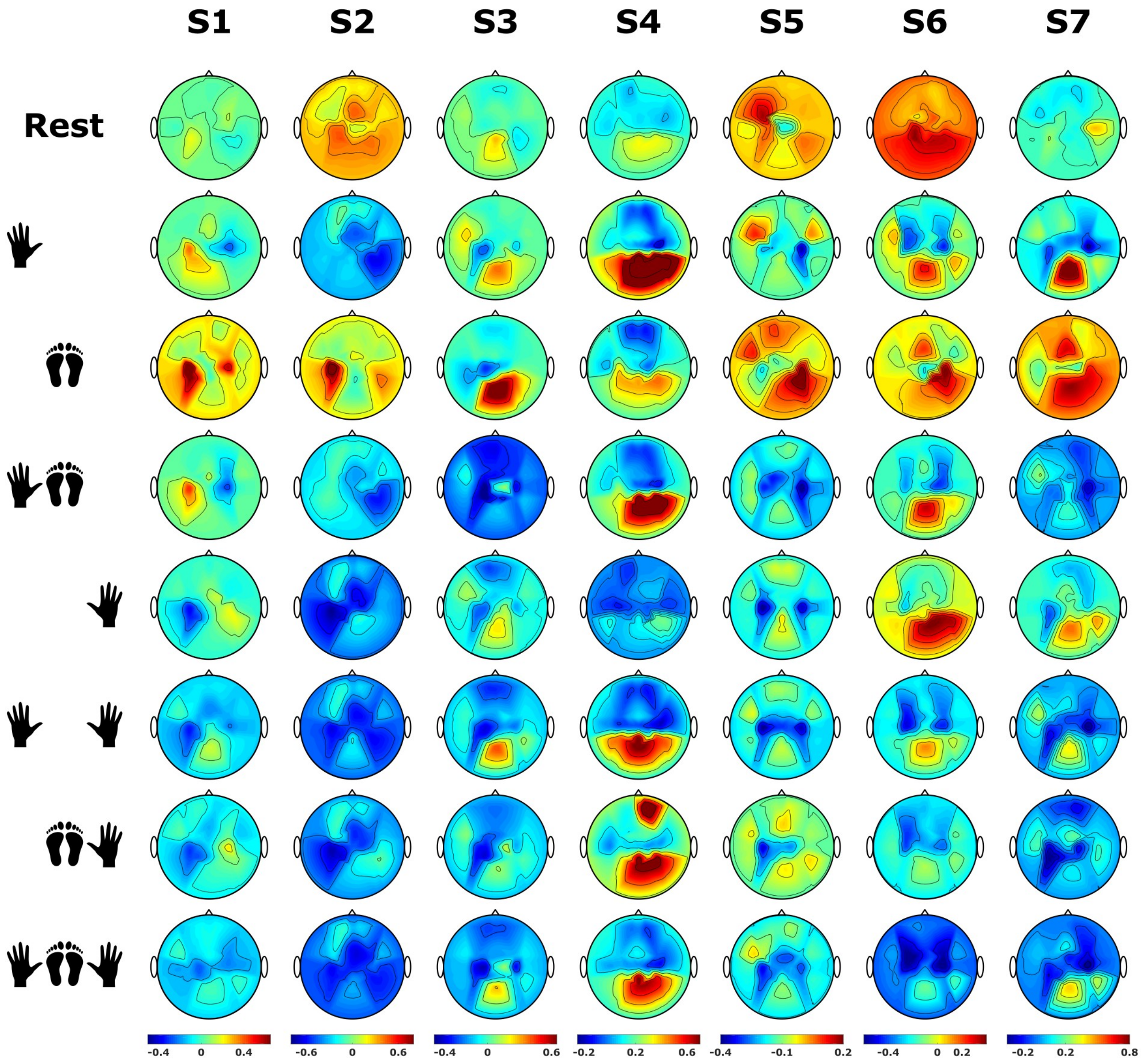

Figure S1: **Time series topographies for all subjects.**

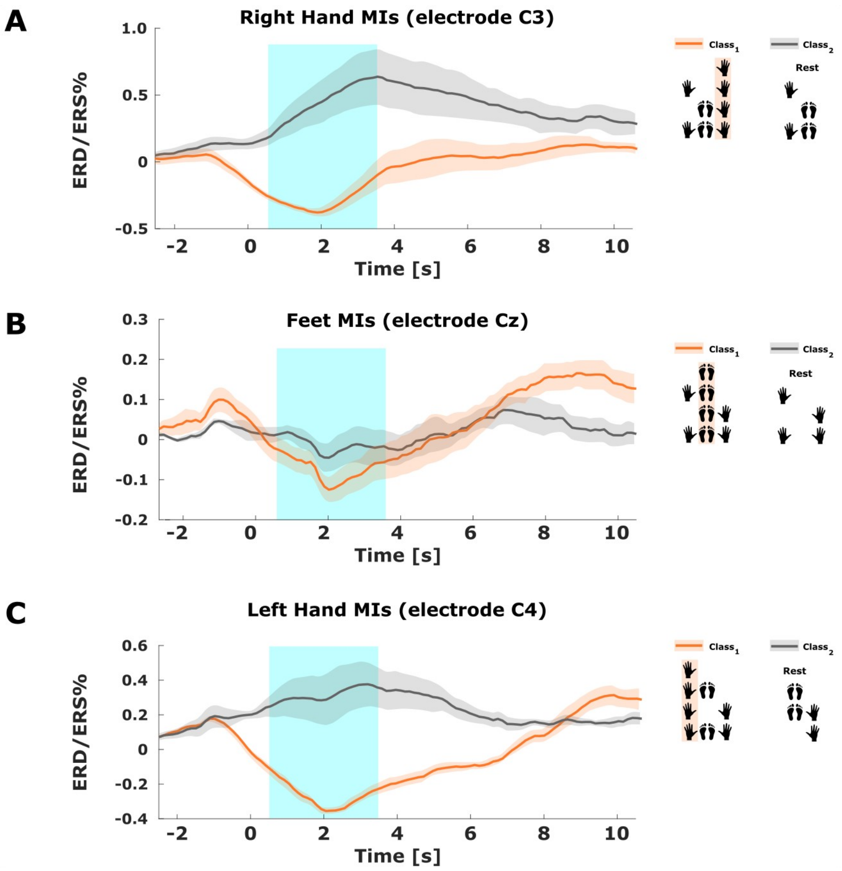

Figure S2: **Modulations over the three main sources for subject 1.** Each plot presents the mean ERS/ERD% patterns of the band-pass filtered EEG trials grouped within class 1 and class 2 for each one the three modules in the CM2CMI method (solid lines). The shaded regions represent the standard errors of the mean, and the blue box within [0.5-3.5 s] indicates the time window that was considered for classification. (A) ERS/ERD% patterns in electrode C4 for class 1, i.e., MIs including the left hand (orange line), and for class 2, i.e., MIs that do not include the left hand (gray line). (B) ERS/ERD% patterns in electrode Cz for class 1, i.e., MIs including the feet (orange line), and for class 2, i.e., MIs that do not include the feet (gray line).(C) ERS/ERD% patterns in electrode C3 for class 1, i.e., MIs including the right hand (orange line), and for class 2, i.e., MIs that do not include the right hand (gray line).

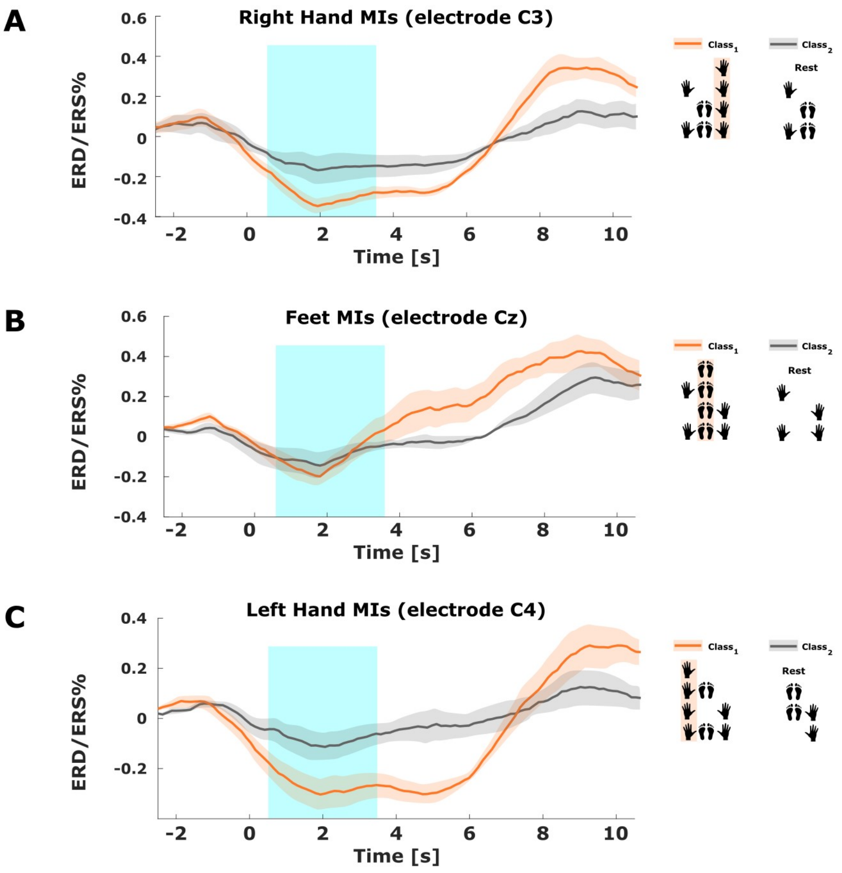

Figure S3: **Modulations over the three main sources for subject 3.** Each plot presents the mean ERS/ERD% patterns of the band-pass filtered EEG trials grouped within class 1 and class 2 for each one the three modules in the CM2CMI method (solid lines). The shaded regions represent the standard errors of the mean, and the blue box within [0.5-3.5 s] indicates the time window that was considered for classification. (A) ERS/ERD% patterns in electrode C4 for class 1, i.e., MIs including the left hand (orange line), and for class 2, i.e., MIs that do not include the left hand (gray line). (B) ERS/ERD% patterns in electrode Cz for class 1, i.e., MIs including the feet (orange line), and for class 2, i.e., MIs that do not include the feet (gray line).(C) ERS/ERD% patterns in electrode C3 for class 1, i.e., MIs including the right hand (orange line), and for class 2, i.e., MIs that do not include the right hand (gray line).

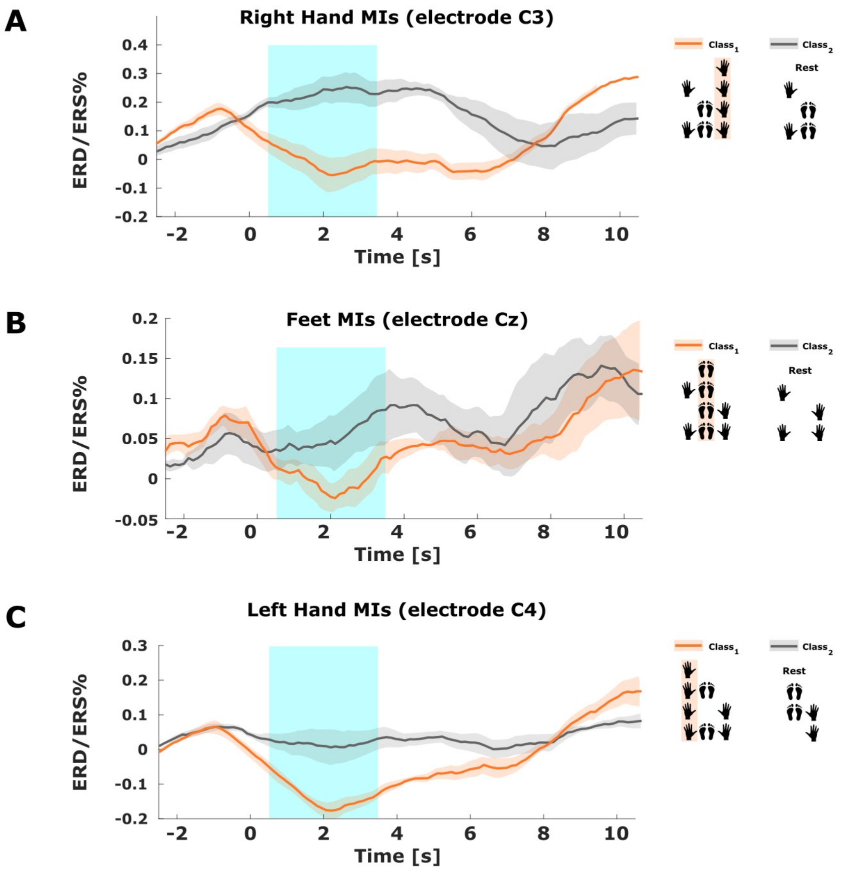

Figure S4: **Modulations over the three main sources for subject 4.** Each plot presents the mean ERS/ERD% patterns of the band-pass filtered EEG trials grouped within class 1 and class 2 for each one the three modules in the CM2CMI method (solid lines). The shaded regions represent the standard errors of the mean, and the blue box within [0.5-3.5 s] indicates the time window that was considered for classification. (A) ERS/ERD% patterns in electrode C4 for class 1, i.e., MIs including the left hand (orange line), and for class 2, i.e., MIs that do not include the left hand (gray line). (B) ERS/ERD% patterns in electrode Cz for class 1, i.e., MIs including the feet (orange line), and for class 2, i.e., MIs that do not include the feet (gray line).(C) ERS/ERD% patterns in electrode C3 for class 1, i.e., MIs including the right hand (orange line), and for class 2, i.e., MIs that do not include the right hand (gray line).

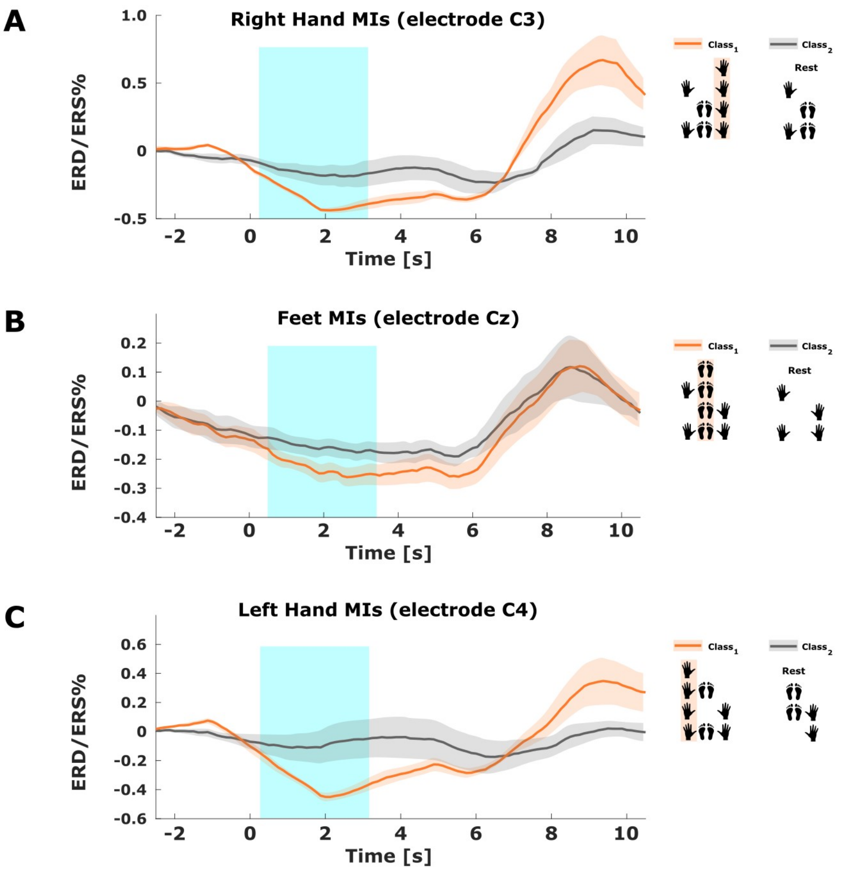

Figure S5: **Modulations over the three main sources for subject 5.** Each plot presents the mean ERS/ERD% patterns of the band-pass filtered EEG trials grouped within class 1 and class 2 for each one the three modules in the CM2CMI method (solid lines). The shaded regions represent the standard errors of the mean, and the blue box within [0.5-3.5 s] indicates the time window that was considered for classification. (A) ERS/ERD% patterns in electrode C4 for class 1, i.e., MIs including the left hand (orange line), and for class 2, i.e., MIs that do not include the left hand (gray line). (B) ERS/ERD% patterns in electrode Cz for class 1, i.e., MIs including the feet (orange line), and for class 2, i.e., MIs that do not include the feet (gray line).(C) ERS/ERD% patterns in electrode C3 for class 1, i.e., MIs including the right hand (orange line), and for class 2, i.e., MIs that do not include the right hand (gray line).

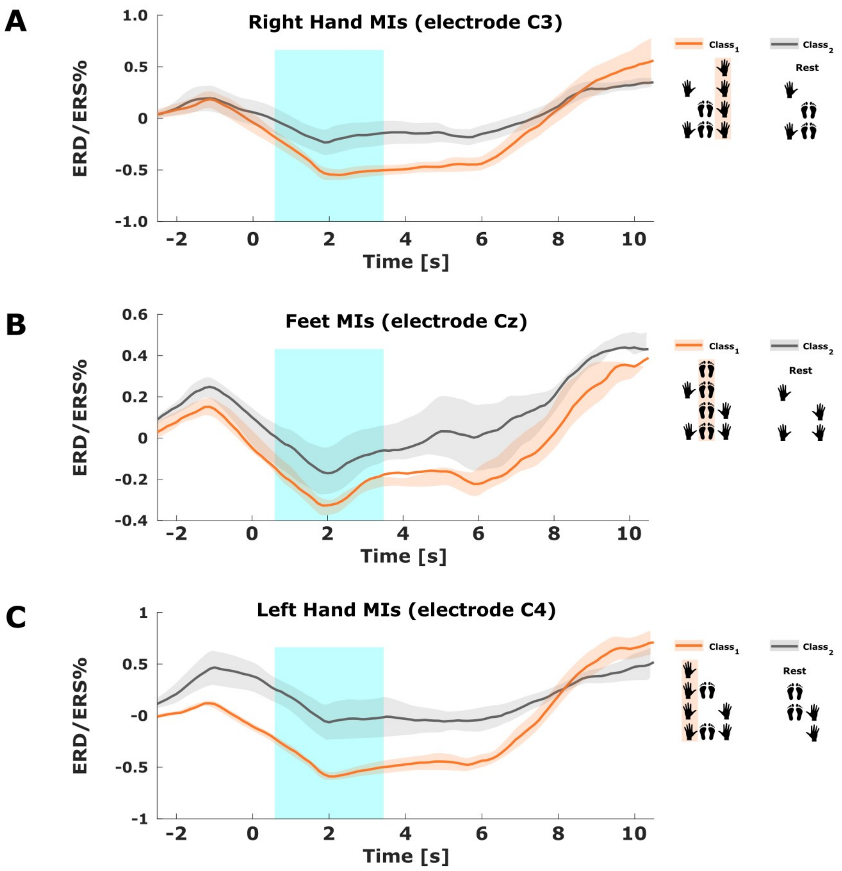

Figure S6: **Modulations over the three main sources for subject 6.** Each plot presents the mean ERS/ERD% patterns of the band-pass filtered EEG trials grouped within class 1 and class 2 for each one the three modules in the CM2CMI method (solid lines). The shaded regions represent the standard errors of the mean, and the blue box within [0.5-3.5 s] indicates the time window that was considered for classification. (A) ERS/ERD% patterns in electrode C4 for class 1, i.e., MIs including the left hand (orange line), and for class 2, i.e., MIs that do not include the left hand (gray line). (B) ERS/ERD% patterns in electrode Cz for class 1, i.e., MIs including the feet (orange line), and for class 2, i.e., MIs that do not include the feet (gray line).(C) ERS/ERD% patterns in electrode C3 for class 1, i.e., MIs including the right hand (orange line), and for class 2, i.e., MIs that do not include the right hand (gray line).

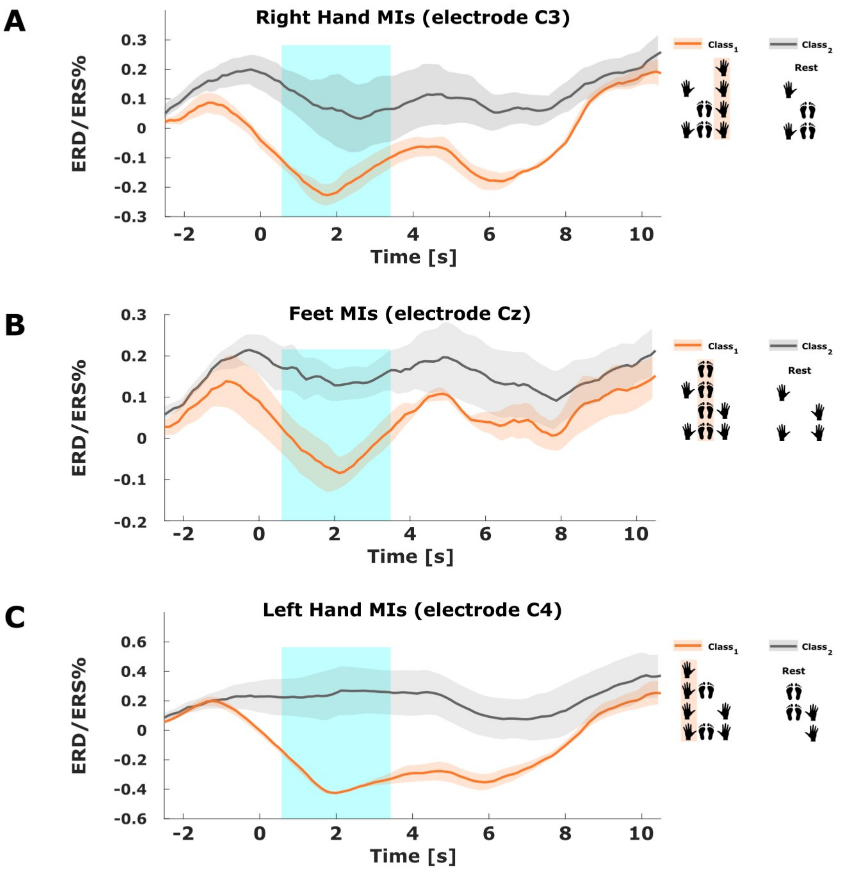

Figure S7: **Modulations over the three main sources for subject 7.** Each plot presents the mean ERS/ERD% patterns of the band-pass filtered EEG trials grouped within class 1 and class 2 for each one the three modules in the CM2CMI method (solid lines). The shaded regions represent the standard errors of the mean, and the blue box within [0.5-3.5 s] indicates the time window that was considered for classification. (A) ERS/ERD% patterns in electrode C4 for class 1, i.e., MIs including the left hand (orange line), and for class 2, i.e., MIs that do not include the left hand (gray line). (B) ERS/ERD% patterns in electrode Cz for class 1, i.e., MIs including the feet (orange line), and for class 2, i.e., MIs that do not include the feet (gray line).(C) ERS/ERD% patterns in electrode C3 for class 1, i.e., MIs including the right hand (orange line), and for class 2, i.e., MIs that do not include the right hand (gray line).

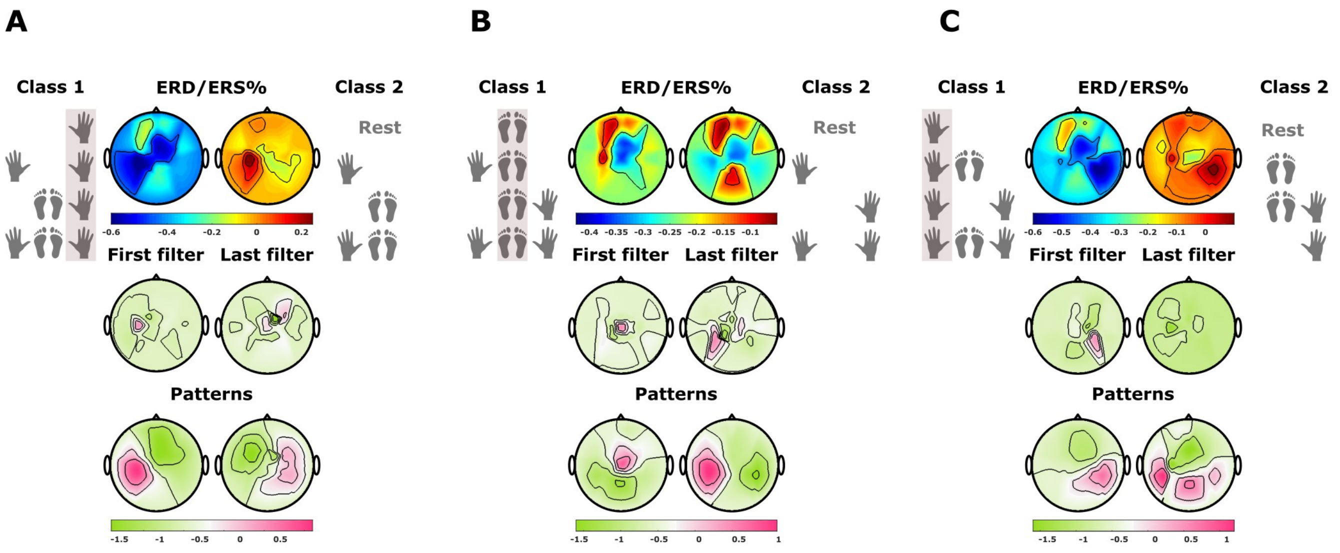

Figure S8: **CSP Analysis for subject 2.** (A) The top topographies show the ERD/ERS% mean values over the selected time window [0.5-3.5 s] for class 1 (not including the right hand) and class 2 (including the right hand). The topographies in the bottom show the first and last filters of the CSP matrix trained over both classes, and the corresponding patterns. The same illustration is presented in (B) for the MIs excluding/including the use of the feet, and in (C) for the MIs excluding/including the use of the left hand.

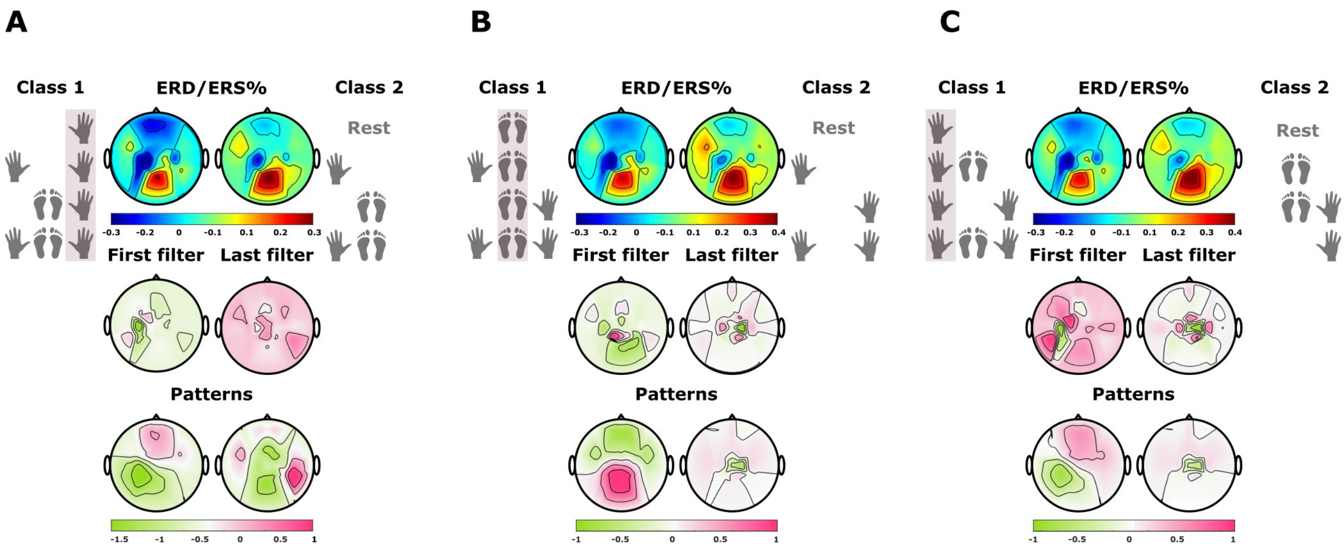

Figure S9: **CSP Analysis for subject 3.** (A) The top topographies show the ERD/ERS% mean values over the selected time window [0.5-3.5 s] for class 1 (not including the right hand) and class 2 (including the right hand). The topographies in the bottom show the first and last filters of the CSP matrix trained over both classes, and the corresponding patterns. The same illustration is presented in (B) for the MIs excluding/including the use of the feet, and in (C) for the MIs excluding/including the use of the left hand.

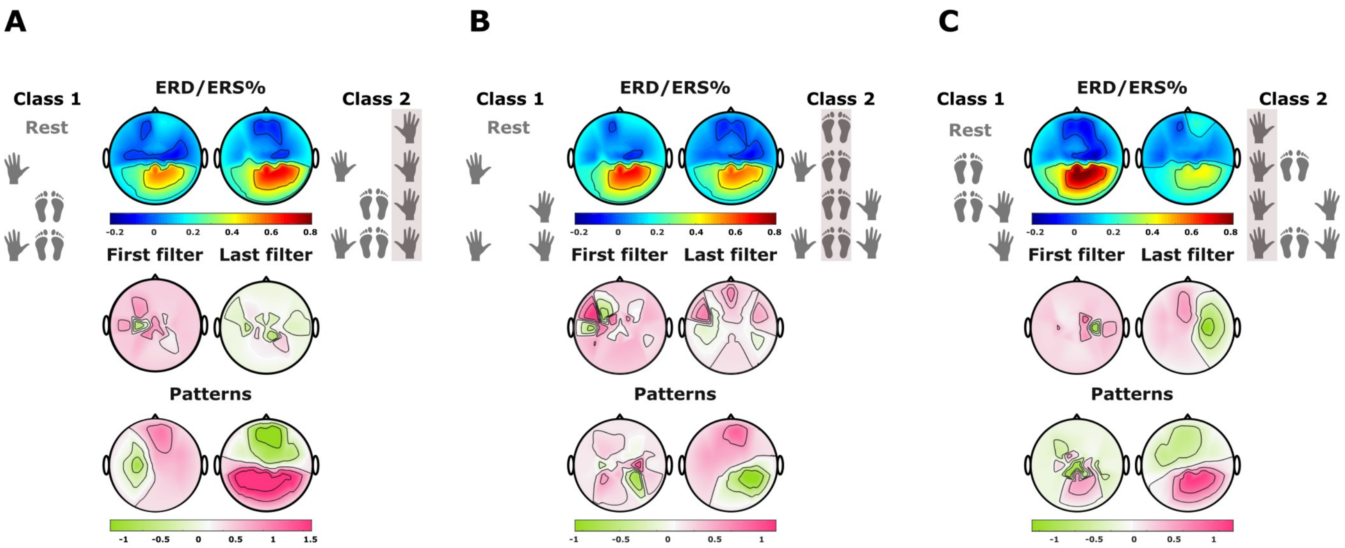

Figure S10: **CSP Analysis for subject 4.** (A) The top topographies show the ERD/ERS% mean values over the selected time window [0.5-3.5 s] for class 1 (including the right hand) and class 2 (not including the right hand). The topographies in the bottom show the first and last filters of the CSP matrix trained over both classes, and the corresponding patterns. The same illustration is presented in (B) for the MIs excluding/including the use of the feet, and in (C) for the MIs excluding/including the use of the left hand.

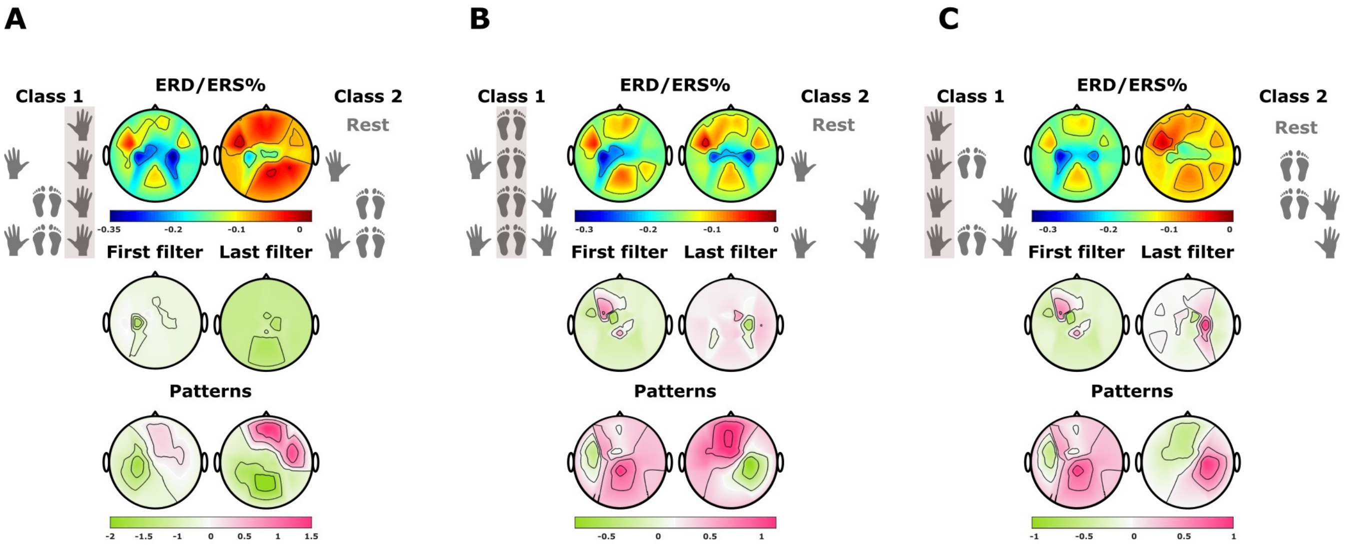

Figure S11: **CSP Analysis for subject 5.** (A) The top topographies show the ERD/ERS% mean values over the selected time window [0.5-3.5 s] for class 1 (not including the right hand) and class 2 (including the right hand). The topographies in the bottom show the first and last filters of the CSP matrix trained over both classes, and the corresponding patterns. The same illustration is presented in (B) for the MIs excluding/including the use of the feet, and in (C) for the MIs excluding/including the use of the left hand.

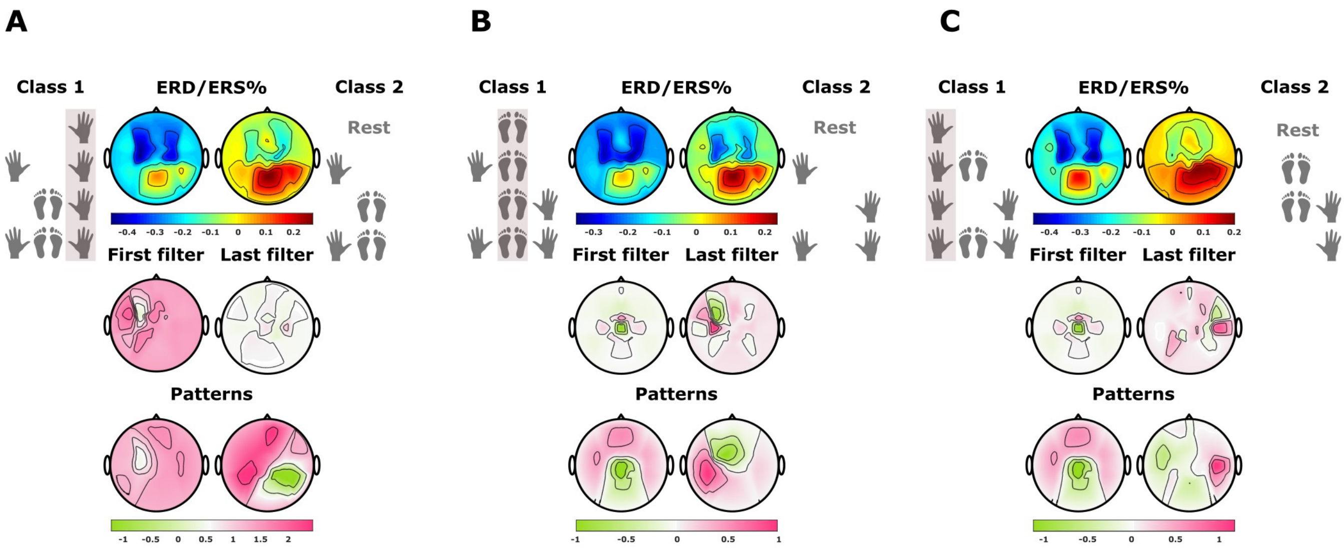

Figure S12: **CSP Analysis for subject 6.** (A) The top topographies show the ERD/ERS% mean values over the selected time window [0.5-3.5 s] for class 1 (not including the right hand) and class 2 (including the right hand). The topographies in the bottom show the first and last filters of the CSP matrix trained over both classes, and the corresponding patterns. The same illustration is presented in (B) for the MIs excluding/including the use of the feet, and in (C) for the MIs excluding/including the use of the left hand.

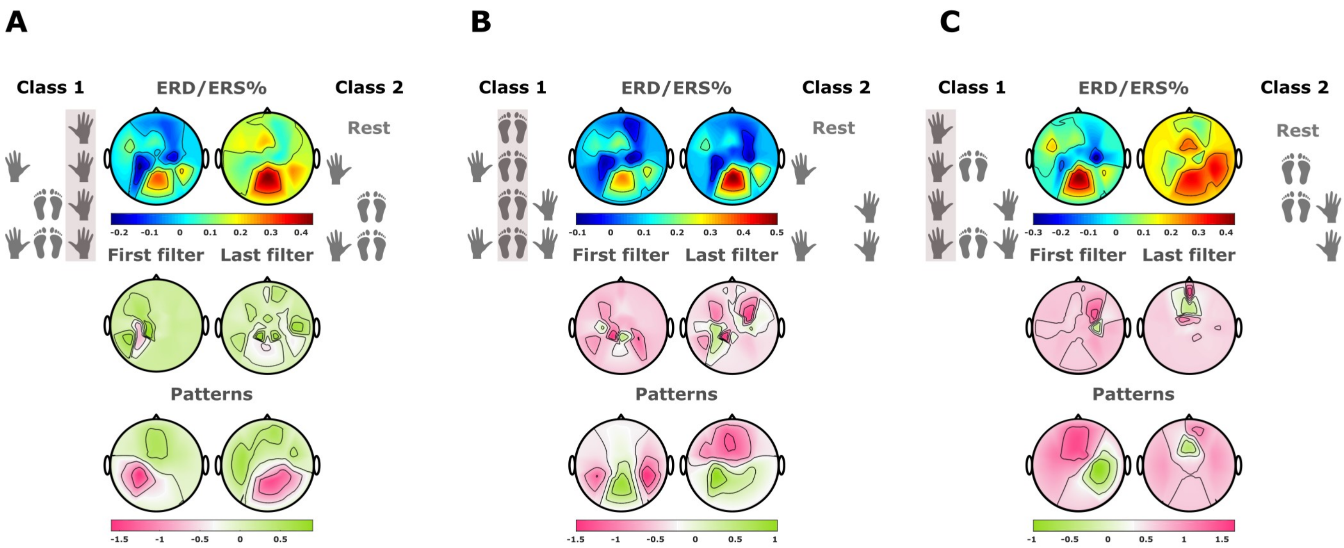

Figure S13: **CSP Analysis for subject 7.** (A) The top topographies show the ERD/ERS% mean values over the selected time window [0.5-3.5 s] for class 1 (not including the right hand) and class 2 (including the right hand). The topographies in the bottom show the first and last filters of the CSP matrix trained over both classes, and the corresponding patterns. The same illustration is presented in (B) for the MIs excluding/including the use of the feet, and in (C) for the MIs excluding/including the use of the left hand.

Table S1: Classification accuracies using SVM.

Subject	MC2CMI	MC2SMI	PW	OVA
1	**47.92**	44.58	40.42	32.08
2	**80.63**	76.88	78.75	70.63
3	47.19	**48.13**	44.69	38.75
4	35.0	**37.5**	29.69	33.12
5	**46.56**	43.13	37.5	35.63
6	**57.5**	**57.5**	51.25	49.69
7	61.88	**63.12**	59.38	57.19
AVG	**53.81±2.58%**	52.98±2.95%	48.81±2.75%	45.3±2.83%

Performance across all subjects after applying a 10-fold-cross-validation procedure to assess the MC2CMI, together with the fast training version denoted as MC2SMI that was considered to optimize calibration times by training only over single MIs. We also present the results generated by the Pair-Wise (PW), and the One-versus-All approaches. All methods were applied over the same band-pass filtered EEG data to provide a fair comparison. The mean average across subjects is presented together with the standard error of the mean.

Table S2: Classification accuracies using Decision Trees.

Subject	MC2CMI	MC2SMI	PW	OVA
1	**45.83**	36.67	38.75	31.67
2	**74.69**	64.38	73.75	60.94
3	39.69	39.06	**41.56**	33.75
4	34.06	**34.38**	31.25	25.62
5	**43.13**	41.25	39.06	34.69
6	**49.09**	43.75	45.31	39.37
7	52.5	40.94	**55.63**	46.88
AVG	**48.42±3.3%**	44.35±3.15%	46.47±3.34%	38.99±3.13%

Performance across all subjects after applying a 10-fold-cross-validation procedure to assess the MC2CMI, together with the fast training version denoted as MC2SMI that was considered to optimize calibration times by training only over single MIs. We also present the results generated by the Pair-Wise (PW), and the One-versus-All approaches. All methods were applied over the same band-pass filtered EEG data to provide a fair comparison. The mean average across subjects is presented together with the standard error of the mean.
